# A comprehensive evaluation of the ecological status of Wadi Mariout ponds, Egypt

**DOI:** 10.1038/s41598-025-97129-6

**Published:** 2025-04-30

**Authors:** Alaa I. Khedr, Mohamed H. Abdo, Radwan G. Abd Ellah, Shaimaa M. Ibrahim, Eman I. Abdel-Aal, Howayda H. Abd El-Hady, Nehad Khalifa, Heba E. A. Elsebaie, Amal A. Othman, Salem G. Salem, Mohamed E. Goher

**Affiliations:** National Institute of Oceanography and Fisheries, NIOF, Cairo, Egypt

**Keywords:** Wadi Mariout, Carlson trophic state index, Water quality index, Bio-polymeric particulate organic carbon, Biotic community, Diversity index, Environmental sciences, Limnology

## Abstract

The present study is the first pioneer study on the ecological status of the Wadi Mariout ponds in the southern part of the Mediterranean Sea, Egypt. The physicochemical variables referred to brackish water (salinity: 12.28–16‰). The WQI indicated a lower water quality status in the western basin than the eastern one. The average TSI values (64.61 and 63.52) refer to high ecological productivity and poor water quality. However, the Arithmetic Water Quality Index (Ar-WQI) indicates the excellent water quality of the eastern pond for aquatic life, while the water quality of the western pond varies from good to very poor. The predominant groups’ species and low biodiversity indices of phytoplankton (1.29–2.2), zooplankton (1.23–1.93), and macroinvertebrates (0.45–1.85), as well as the biochemical composition of phytoplankton, reveal a high protein-carbohydrate ratio (> 11), indicating eutrophication conditions. Also, the biotic measurements show that the phytoplankton (70.7 × 10^6^ Ind.l^−1^) and zooplankton (23.23 × 10^5^ Ind.m^−3^) communities were more diverse and flourished in the western basin. Nevertheless, the macrobenthic invertebrates varied more in the eastern basin, 31,284 org.m^−2^, in compared to 5750 org.m^−2^ in the western one. The phytoplankton community was dominated by Chlorophyta and Cyanobacteria (79.5–93%), zooplankton by Rotifera (62.5–86.3%), and macrobenthos by Mollusca (62%) at the western basin and Annelida (38%) at the eastern one. The total bacteria count and fecal coliform value were rare in Wadi Mariout water. On the other hand, the sediment samples have more total coliform (15–3348 cfug^−1^) than the water (0–12 MPN/100). Wadi Mariout ponds, similar to Egyptian Delta lakes, suffer from the eutrophication phenomenon, which must be treated by controlling the feeding wastewater supplies of the ponds. The study sheds light on the current environmental status of Wadi Mariout lakes, serving as the first comprehensive study to assess water quality, record and classify biodiversity, and provide a precise assessment of the water body’s situation. This will facilitate the effective management of these important Egyptian lakes in the future.

**Supplementary Information:**

The online version contains supplementary material available at 10.1038/s41598-025-97129-6.

## Introduction

Water quality assessment plays a crucial role in resource management and contamination control^1^. Water quality contamination is a serious and common issue worldwide for numerous reasons, including natural processes, anthropogenic activities, and overdevelopment^2,3^. Understanding the physicochemical properties is essential for several applications concerning the lake’s water. Knowledge of nutrient salt content allows us to predict lake eutrophication^4^. Any significant changes in water quality will usually be disruptive to the ecosystem^5^. Thus, regular water quality assessments are necessary to evaluate water quality for ecosystem health, industrial, agricultural, and residential use^6^. Assessing water quality when dealing with large samples that contain varying quantities of multiple factors can be challenging. Traditional techniques for evaluating the quality of water rely on comparing the tested and determined variable’s values with the most recent recommendations. Thus, techniques that significantly minimize the amount of data and streamline the depiction of the state of water quality are known as water quality indices^7^. On the other hand, the bacterial community has long been a vital part of the aquatic environment since it helps to ensure the ecosystem’s continuity. Researchers are interested in the diversity of aquatic bacteria because of their link to ecosystem pollution. Water contamination is a significant threat to the environment since it negatively impacts both aquatic ecosystem biodiversity and human health^8^.

In aquatic environments, phytoplankton are excellent biological indicators of water quality. Their abundance, species diversity, and composition are used to judge the biological integrity of the water system^9^. Also, they have a critical role in the ecosystem’s primary productivity, nutrient cycling, and food webs^10–12^. Phytoplankton provides many valuable phytonutrients and biologically active compounds, especially proteins, carbohydrates, and lipids^13^. Biochemical components of phytoplankton can provide important quantitative and qualitative information about the food available for the food chain^14^. Differences in phytochemical values can lead to differences in nutritional qualities for potential consumers^14,15^. Phytoplankton is firmly the most important part of particulate biopolymeric organic carbon (BPC) in surface waters^16^. Mostly derived from phytoplankton, particulate organic matter (POM) serves as a crucial food source, connecting primary producers to herbivores^17–19^. Researchers have considered the biomolecular composition of phytoplankton as an indicator of the physiological responses to environmental changes^20,21^, which in turn correlates with their nutritional quality for higher trophic consumers^22^. Zooplankton plays a critical role in regulating aquatic productivity, occupying a focal position in the food chain, and serving as good bioindicators of environmental status over time^23,24^. Using zooplankton is a valuable tool to determine the water quality status as one of the Biological Quality Elements (BQE) within the Water Framework Directive (WFD) metrics^25^.

Sediments, varying from gravel to fine mud, blanket the majority of aquatic environments. These sediments can be uniform or mixed in grain size and originate from both biological and geological sources, creating diverse habitats^26^. Numerous aquatic species inhabit these sediments, where microbial activity drives nutrient regeneration and cycling, crucial processes of the entire water body. Furthermore, the dynamic nature of sediments, influenced by factors like wet-dry cycles and salinity gradients, fosters high biodiversity^26^. Sediments are crucial for determining the variable, composition, and ultimately, the quality of aquatic habitats, which may be used to determine the quality or health of the water body^27^. Sediment is considered a reservoir of pathogenic bacteria and exhibits potential health hazards from probable resuspension to water^28,29^. Benthic invertebrates, known as macroinvertebrates, exhibit a wide range of species^30^ and serve as valuable indicators of water quality due to their ability to respond to cumulative pressures over time, reflecting the changing environmental circumstances. The impact of different contaminants on the macrobenthic community can be evaluated by analyzing diversity, the tolerance levels of recognized organisms, and, in certain instances, the relative abundances and feeding habits^31^. Macroinvertebrates hold an intermediate position in the food chain of the majority of aquatic habitats. Macrobenthos inhabit many ecosystems and are found close to the top layer of silt^32^. Benthic macroinvertebrates and benthic algae are valuable indicators for assessing the ecological condition of water bodies^33–35^. The impoverished water quality has a detrimental impact on all the living organisms inside an aquatic ecosystem^36^. Benthic invertebrates play a crucial role in evaluating the overall health of ecosystems and are essential for the productivity of lakes. They convert organic material into biomass and contribute to the nutritional needs of fish^37^ Benthic invertebrates display significant diversity and abundance based on their environments, and they are crucial for transmitting energy to higher trophic levels. Benthic invertebrates have significant functional impacts on fish productivity^38,39^. The primary determinants influencing the structure and dispersion of the benthic ecosystem are the characteristics of the underlying sediment, the salinity of the soil, and the content of organic matter, in addition to the abundance of stocked fish populations^40,41^.

Egypt has many lakes distributed from north to south. Some are natural, while others are artificial. In northern Egypt, south of the Mediterranean Sea, five well-known lakes stretch along the Delta coast toward Sinai. These five lakes, from west to east, are Mariout, Idku, Burullus, Manzala, and Bardawil. West of Lake Mariout, Wadi Mariout Lake is located^6,42–44^. Wadi Mariout Lake is one of the remaining parts of the ancient Lake Mareotis and is not mentioned in the scientific literature. It differs from the famous Mariout Lake, also considered a remnant of the Lake Mareotis. To date, there have been rare investigations of biodiversity and accurate characterization of the different ecosystems in Wadi Mariout ponds. Wadi Mariout Lake separates from the principal portion of Mariout Lake by several causeways, canals, and roadways. It is a closed basin without a continuous water supply, receiving input from groundwater seepage, agricultural runoff, and precipitation^45^. Significant portions of this basin remain arid for much of the year. Wadi Mariout Lake exhibits greater stability than Mariout Lake due to its historical distance from the influence of Nile sediments, and its size has remained relatively constant^45^. According to Hassan and Badran^45^, the Wadi Mariout ponds have not been exposed to the discharge of industrial wastes and have experienced minimal urban activities, consequently, these lakes serve as a valuable reference location for assessing the sediment contamination levels of other lakes in Egypt, especially the Norther Delta lakes. They verified the water quality situation of the lake and validated its significance as an indicator and comparative model for coastal lakes in the south Mediterranean^45^. Thus, the primary goal of this study is to (1) Identify the current environmental status of two basins of the Wadi Mariout using the arithmetic water quality index (Ar-WQI) to safeguard aquatic life; (2) Examine the eutrophication state using the Carlson trophic state index (CTSI); (3) Study the phytoplankton community and how it is correlated with biotic and abiotic factors that affect phytoplankton growth and abundance; (4) Evaluate the species composition, density, and distribution of zooplankton; (5) Evaluate the nutritional value of phytoplankton and the quantity and quality of particulate biopolymeric organic carbon (BPC) concerning the biological environmental conditions; (6) Assess the bacterial diversity in water and sediment; (7) Study the biodiversity and abundance of macrobenthic invertebrates in Wadi Mariout Pond and examine the variation in faunal assemblages in the eastern and western basins. In general, the study sheds light on the current environmental status of Wadi Mariout lakes as the first comprehensive study to assess water quality and recording and classification of the biodiversity in the Wadi Mariout Pond that will provide a precise assessment of the water body’s situation. This will facilitate effective management of the important Egyptian lakes in the future.

## Results and discussion

### Water results

#### Physico-chemical variables

The physical and chemical characteristics of the two basins of Wadi Mariout are presented in Table 1. The water temperature influences the chemical and biological processes of the water bodies^46^. Water temperature fluctuated between 27.99 °C and 28.15 °C in the eastern basin and from 28.51 °C to 28.74 °C in the western basin, with significant differences between the sites (*p* < 0.001, *n* = 30). The water temperature was within the permissible range for aquatic fish (8–28 °C)^47^. Drastic temperature changes can be fatal to fish^47^. The Wadi Mariout basins were highly turbid; the transparency fluctuated between 90 cm at S1 and 115 cm at S2 in the eastern basin, while it ranged from 80 cm at S9 and S10 to 130 cm at S6 in the western basin. pH is a valuable tool that affects the overall aquatic environment. pH fluctuated in a narrow range with significant differences, between 7.88 and 7.94 in the eastern basin and between 8.01 and 8.07 in the western basin. Electrical conductivity (EC) determines the concentration of dissolved electrolyte ions in the water. Significant changes in pH and EC may be indicators for different pollution discharges. EC showed a significant variation between the two basins at *p* < 0.01. EC fluctuated between 21.96 and 22.86 mS/cm with average values of 22.58 ± 0.36 mS/cm in the eastern basin and between 17.54 and 20.08 with average values of 18.81 ± 1.19 mS/cm in the western basin. The eastern basin appears to be more saline than the western basin. Salinity varied from 15.37‰ at S 5 to 16.00‰ at S1 in the eastern basin and from 12.28‰ at S 10 to 14.06‰ at S7 in the western basin, with significant differences between the two basins at *P* < 0.01. It has been found that salinity is positively related to EC (*r* = 0.999, *P* < 0.001). This could mean that EC is sensitive to changes in the amounts of dissolved salts at different sites^47^. The current salinity results were within the ranges noted by Burullus Lake and Edku Lake by Al-Afify et al.^43^ and Radwan et al.^48^, respectively. Dissolved Oxygen (DO) levels in the two basins were higher than the acceptable level (4 mg/l) for the protection of aquatic life^49^. They fluctuated within 4.79 ± 0.21 and 6.48 ± 0.71 mg/l in the eastern and western basins, respectively. The western basin appears to be significantly more oxygenated than the eastern basin at *p* < 0.05 (Table 1), which may be due to higher phytoplankton abundance, which enhanced oxygen throughout photosynthesis activity. Biological oxygen demand (BOD) and chemical oxygen demand (COD) may refer to water pollution by organic matter from industrial and agricultural wastes and treatment plant effluents^50^. The BOD concentrations were at the acceptable level of < 6 mg/l reported for aquatic life by CCME^49^. It significantly fluctuated between (2.55–3.16 mg/l) and (3.14–5.44 mg/l), with average values of 2.99 ± 0.29 and 4.64 ± 0.91 mg/l in the eastern and western basins, respectively. COD fluctuated similarly between the two basins in a narrow range, with average values of 12.16 ± 1.04 and 11.60 ± 2.33 mg/l in the eastern and western basins, respectively. According to CCME^49^, COD was just above the acceptable limit for aquatic life (Table 1).

Carbonate, bicarbonate, and total alkalinity fluctuated in the ranges of (7.5–15), (125.05–164.7), and (152.5–165) mg/l in the eastern basin and (7.5–12), (146.4–161.65), and (142.5–162.5) mg/l in the western basins, respectively. Chloride and sulfate ions are the major anions in the Wadi Mariout basins. As the eastern basin is more saline than the western one, Cl^−^ and SO_4_^2−^ are significantly higher in the eastern basin at (*P* < 0.001 and *P* < 0.05, respectively). Cl^−^ and SO_4_^2−^ had an average concentration of 6.04 ± 0.17 g/l and 3.97 ± 0.14 g/l, respectively, in the eastern basin and 4.88 ± 0.34 and 3.77 ± 0.1 g/l, respectively, in the western one. The average concentrations of the major cations: Na, K, Ca, and Mg were found to be 4.66 ± 0.16, 0.2 ± 0.02, 0.57 ± 0.03, and 0.38 ± 0.01 g/l, respectively, in the eastern basin and were 4.13 ± 0.14, 0.15 ± 0.01, 0.54 ± 0.02, and 0.33 ± 0.02 g/l, respectively in the western basin. The Na, K, and Mg results show significant differences between the two basins at *P* < 0.01. The predominant major cations in the Wadi Mariout ponds are Na > Ca > Mg > K.

The Wadi Mariout Pond showed brackish water at all the sites with salinity values between 12.28‰ and 16‰. This pattern is different from the other Egyptian north lakes that showed brackish water at some sites and saline water at other sites. However, it showed lower turbidity than Mariout^51^, Manzala^4,52^, and Burullus lakes^47^, but depicted similar transparency to Edku Lake^48^. On the other hand, the DO content was lower in Wadi Mariout than in Manzala^52^, Burullus^4–6,47^, and Edku Lake^48^ which may be due to the closed nature of the pond with lower renewable water (Table 2).

#### Nutrient salts and chlorophyll-*a*

Compounds comprising N-, P-, or Si in either available or unavailable forms are included in the nutritional salts (NO_2_^−^, NO_3_^−^, NH_4_^+^, PO_4_^3−^, and SiO_4_^−^)^47^. They are crucial to the productivity of aquatic environments since they serve as fish and phyto- and zooplankton’s feeding chains. Nitrosomonas bacteria, which are autotrophic, utilize oxygen and ammonia to produce nitrite, an intermediary product of aerobic nitrification. Fertilizer runoff can directly cause nitrate (NO_3_^−^) to enter the water body^11^. Both the ionized form (NH_4_^+^) and the hazardous non-ionized form (NH_3_) are included in total ammonia. The average levels of the nutritional salts; SiO_4_^−^, NH_4_^+^, NO_2_^−^, NO_3_^−^ and PO_4_^3−^ were 9.86 ± 1.63 mg/l, 428.46 ± 94.53 µg/l, 7.76 ± 1.34 µg/l, 21.88 ± 6.79 µg/l, and 76.79 ± 11.32 µg/l, respectively in the eastern basin; and were 8.19 ± 2.36 mg/l, 558.45 ± 290.75 µg/l, 28.56 ± 15.0 µg/l, 417.86 ± 397.46 µg/l, and 55.32 ± 10.43 µg/l, respectively in the western basin. The distribution of silicate concentrations between the different sites may be correlated with the nature of the lake’s sediment, which consists of sand^53,54^. Nitrite, nitrate, and phosphate were significantly different between the two basins (*p* < 0.05, *p* < 0.01, *p* < 0.05, respectively, *n* = 30). Ammonia represented 93.42, and 58.54% of DIN in the eastern and western basins, respectively. High levels of ammonia may be lethal for fish and other aquatic organisms^55^. However, ammonia, nitrite, and nitrate concentrations were less than the threshold levels of 1370, 60, and 2930 µg/l, respectively, reported for aquatic life by CCME^49^ (Table 1). Total Phosphorus (TP) fluctuated between 142.49 and 172.25 µg/l in the eastern basin and between 141.07 and 157.37 µg/l in the western basin. Al-afify et al.^43^ stated that the presence of an overabundance of nitrogen and phosphorus in the water of Burullus Lake can lead to the formation of algal blooms, which subsequently have a detrimental effect on the overall water quality. This study declared lower ammonia, nitrite, nitrate, phosphate, TP, and silicate concentrations at the two Wadi Mariout basins than that was recorded at Mariout Lake in a previous work by El-Degwy^51^. In addition, phosphate, ammonia, nitrite, and nitrate showed lower concentrations than those recorded in Burullus Lake by Zaghloul et al.^42^, Table 2.

Chl-a is a common environmental metric of algae, or phytoplankton, biomass development as well as the eutrophication process in oceans, lakes, and reservoirs^15^. It is crucial to the phytoplankton flourishing^16^. Chl-a varied within (10.48–12.97 µg/l) and (8.33–28.11 µg/l) in the eastern and western basins, respectively. Chl-a concentrations were higher than the guideline value of 10 accepted for aquatic life^49^. The positive correlation between Chl-a with nitrite, and nitrate (*r* = 0.662, 0.91, 0.955, *n* = 30, *P* < 0.05), respectively, may indicate the role of nitrite-N and nitrate-N in flourishing the phytoplankton growth and the biological productivity that increased the suspended solids and Chl-a level^56^. The variations in physicochemical parameters along with nutrient salts and Chl-a were outlined in Figs. S1–S16 (in the supplementary file).

**Table 1:** Water quality parameters in Wadi Mariout basins compared to the standard permissible guidelines for aquatic life.

	Eastern Basin				Western Basin				Aquatic live
	Min	Max	Avg	SD	Min	Max	Avg	SD	CCME^49^
Water Temperature (⁰C)	27.99	28.15	28.08	0.07	28.51	28.74	28.61	0.1	8–28
pH	7.88	7.94	7.89	0.03	8.01	8.07	8.03	0.03	6.5-9
Salinity (‰)	15.37	16	15.8	0.25	12.28	14.06	13.17	0.83	
EC (mS/cm)	21.96	22.86	22.58	0.36	17.54	20.08	18.81	1.19	
Trans (cm)	90	115	106	9.62	80	130	103	23.87	
DO (mg/l)	4.64	5.15	4.79	0.21	5.74	7.39	6.48	0.71	> 5.5
BOD (mg/l)	2.55	3.16	2.99	0.29	3.14	5.44	4.64	0.91	< 6
COD (mg/l)	11.2	13.6	12.16	1.04	9.2	14.4	11.6	2.33	7
CO_3_^2−^ (mg/l)	7.5	15	11.4	2.92	7.5	12	9.9	1.71	
HCO_3_^−^ (mg/l)	125.05	164.7	147.01	16.05	146.4	161.65	154.33	5.54	
TA (mg/l)	152.5	165	158.5	4.54	152.5	162.5	159.5	4.11	
Cl^−^ (g/l)	5.77	6.21	6.04	0.17	4.44	5.24	4.88	0.34	0.120
SO_4_ ^2−^ (g/l)	3.78	4.15	3.97	0.14	3.7	3.89	3.77	0.1	
Na (g/l)	4.49	4.89	4.66	0.16	3.98	4.3	4.13	0.14	
K (g/l)	0.19	0.23	0.2	0.02	0.14	0.16	0.15	0.01	
Ca (g/l)	0.54	0.6	0.57	0.03	0.51	0.56	0.54	0.02	
Mg (g/l)	0.36	0.4	0.38	0.01	0.31	0.36	0.33	0.02	
Chl-*a (µg/l)*	10.48	12.97	11.75	0.89	8.33	28.11	16.6	7.33	
SiO_4_ (µg/l)	7.89	11.55	9.86	1.63	5.89	11.46	8.19	2.36	
NH_4_ (µg/l)	334.28	577.97	428.46	94.53	281.78	996.39	558.45	290.75	910*
NO_2_ (µg/l)	6.62	9.72	7.76	1.34	15.01	51.51	28.56	15	60
NO_3_ (µg/l)	14.94	31.23	21.88	6.79	92.58	1048.16	417.86	397.46	2935
PO_4_ (µg/l)	66.3	93.05	76.79	11.32	42.92	69.49	55.32	10.43	
TP (µg/l)	142.49	172.25	152.27	12.06	141.07	157.37	151.7	6.7	
DIN: DIP ratio	5.16	6.65	5.94	0.67	14.98	25.76	18.44	4.56	
TC (cfug^−1^/mL)	0	12	–	–	4	12	6	3	
FC (cfug^−1^/mL)	0	3	–	–	0	3			
FS (cfug^−1^/mL)	0.24 × 10^3^	1.1 × 10^3^	0.93 × 10^3^	0.39 × 10^3^	0.46 × 10^3^	120 × 10^3^	24.5 × 10^3^	53.4 × 10^3^	
TBC at 37 °C (cfug^−1^/mL)	4 × 10^4^	40 × 10^4^	16.6 × 10^4^	17.9 × 10^4^	3.2 × 10^4^	4.2 × 10^4^	3.9 × 10^4^	0.4 × 10^3^	
TBC at 22° C (cfug^−1^/mL)	3.1 × 10^4^	35 × 10^4^	15.4 × 10^4^	16.7 × 10^4^	27 × 10^4^	330 × 10^4^	3.18 × 10^4^	0.27 × 10^3^	

*Australian permissible value depending on temperature and pH value.

#### DIN/DIP ratio

Eutrophication in aquatic environments is governed by a limiting component, either P or N, which can be identified by measuring the dissolved inorganic nitrogen to dissolved inorganic phosphorus (DIN: DIP). Dissolved Inorganic Nitrogen (DIN), is the sum of the concentrations of inorganic nitrogen form (ammonia + nitrite + nitrate). Dissolved Inorganic Phosphate (DIP) is the concentration of available phosphate. The DIN: DIP ratios less than 10 are indicative of nitrogen limiting factors while ratios between 10 and 20 indicate N- and P-limiting variables. Other DIN: DIP ratios larger than 20 are considered to be phosphorus-limited^57^. In the present study, DIN: DIP ranged between 5.16 and 6.65 in the eastern basin, indicative of an N-limiting factor. On the other hand, DIN: DIP showed ratios larger than 10 at most sites of the western basin, suggesting N- and P-co-limited. Only site 6 in the western basin recorded DIN: DIP of 25.76, indicative of being P-limited, which may be attributed to the relative high levels of ammonia and nitrate in the western basin. In comparison, Zaghloul et al.^42^ stated low DIN: DIP ratios (< 10) in Bardawil, Manzala, and Burullus Lakes which were 3.88, 2.48, and 3.86, respectively, confirming N-limiting factors in these lakes. On the other hand, Elsayed et al.^47^ reported high TN: TP ratios of 25.4 in Burullus Lake, inferring it to be P-limited. El Zokm et al.^58^ reported that the nitrogen was the limiting factor in the Mariout Lake water.

#### Carlson trophic state index (CTSI)

**Fig. 1:**
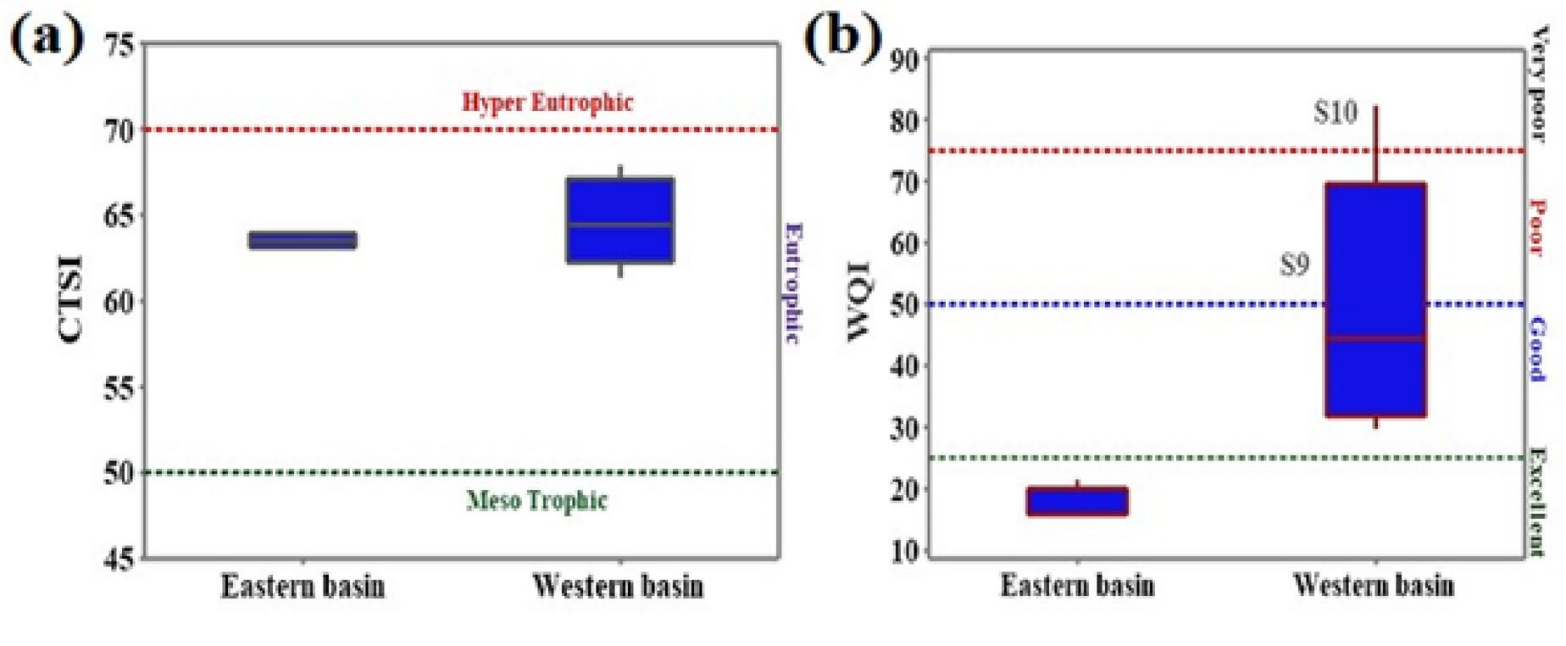
(**a**) CTSI and (**b**) WQI values at the eastern and western basins of Wadi Mariout Pond.

The trophic state index is designed as an indication of the eutrophication status and the water quality of the aquatic system. The lakes may be classified into three classes: oligotrophic, mesotrophic, or eutrophic. Lakes having exceptionally high trophic indices are referred to as hypereutrophic. The higher the CTSI value, the more the eutrophication, the worse the water quality is^59^. The results of CTSI varied in a narrow range between 62.93 and 63.99 in the eastern basin and 61.31–67.88 in the western basin, with no significant differences between the two basins. Based on the CTSI criteria, the two basins showed a state of eutrophic that refers to high ecological productivity and poor water quality (Table S1, in the supplementary file, and Fig. 1a). Inland lakes frequently face eutrophic conditions^60^. The study area’s eutrophic condition is possibly caused by an overabundance of nutrients, which elevates the Chl-*a* concentration. Zaghloul et al.^42^ recorded a CTSI value of 69.73 in Bardawil Lake, which was considered to be eutrophic. Moreover, Manzala and Burullus Lakes were classified as hypereutrophic based on CTSI values of 90.68 and 86.20, respectively^42^. Mahmoud et al.^52^ reported CTSI values between 66 and 80 in the Manzala Lake water, thus varying from a eutrophic state at 33% of samples to a hypereutrophic state at 67% of samples. Both Alprol et al.^44^ and Elsayed et al.^47^ reported that high CTSI values of Burullus Lake between (191.95–200.91) and (73.62–86.1), respectively, inferred a hypereutrophic state and very poor water quality. The poor water quality of the Wadi Mariout basins and the other Egyptian lakes may be attributed to the discharge of sewage water, industrial wastewater, drainage water, and untreated domestic waste into these lakes.

#### Arithmetic water quality index (Ar-WQI)

The WQI summarizes the composite effects of individual parameters in a single value that controls the overall water quality^61^. Ar-WQI values varied between 6.65 and 9.68 in the eastern basin and between 14.26 and 42.04 in the western basin. The Ar-WQI values were less than 25 at all sites of the eastern basin, classified as excellent for aquatic life protection. On the other hand, some sites in the western basin showed WQI values > 25 at Sites 6, 7, and 8, ranked as having good water quality, while S9 showed poor water quality and S10 exhibited very poor water quality for aquatic life protection (Fig. 1b). The WQI results showed that the water quality worsened at the Wadi Mariout basins as we directed from the eastern basin towards the western one, which may be attributed to the impact of the agriculture runoff in the western basin. In comparison, Goher et al.^61^ classified the two Rayan lakes as fair for aquatic life using the Canadian WQI. The present physicochemical measurements and nutrient concentrations of the Wadi Mariout basins are compared with other lakes in Egypt in Table 2.

#### Bacteriological analysis

**Fig. 2:**
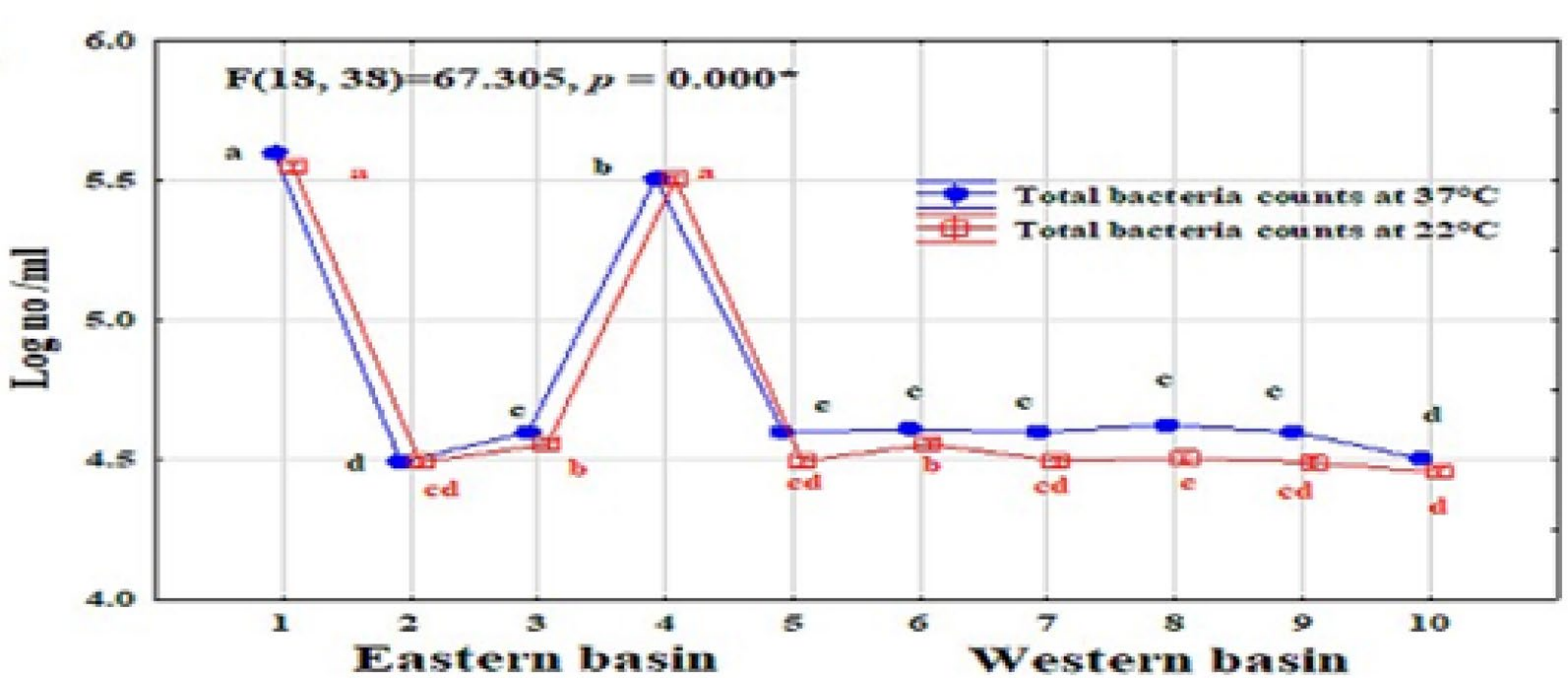
Spatial changes in a microbial load (ANOVA analysis) at the eastern and western basins of Wadi Mariout Pond.

The total bacteria count (TBC) showed different ranges depending on the incubation temperature: at 37 °C (autochthonous), it varied from 3.2 × 10⁴ to 4.0 × 10⁵ cfu mL⁻¹, and at 22 °C (allochthonous), it ranged from 2.9 × 10⁵ to 3.5 × 10⁵ cfu mL⁻¹(Fig. 2). ANOVA analysis indicated significant differences attributed to sites. Among sites, S1 and S4 recorded a particularly high number of TBC counts. ANOVA analysis of bacterial indicators of pollution indicated significant differences attributed to sites (Table S2, in the supplementary file). Total coliform populations were in the range of 0–12 MPN/100 ml, while fecal coliform was detected in only two sites (S5 and S7) that recorded 3 MPN/100 ml. Coliform bacteria showed a significant variation between sites, this variation among sites may be due to aquaculture activity that close to site 1, while in the rest sites may be due to agricultural runoff, wild birds, and or re-suspension of bacteria from the sediment especially in S4 and S5, which recorded highest counts of coliform bacteria in sediment samples. Fecal streptococci show enrichment values in all samples ranging from 2.4 × 10^2^ to 12 × 10^4^ MPN/100 ml. Among sites, S10 recorded the highest values of FS in water. The ratio of FC/FS is considered a qualitative indicator of pollution. Hence, domestic sewage gives ratios exceeding 4.0, whereas a ratio less than 0.7 indicates contamination from animal wastes. The low FC/FS ratio of all samples indicates the non-human source of pollution^5,65^. Where fecal streptococci are mainly derived from livestock and wild animals, agriculture wastewater, and rainwater, this ratio is consistent with the nature of the pond; it is fed by rainfall, agricultural runoff, and groundwater seepage. Survival of bacteria in surface water depends upon several factors, e.g., temperature, salinity, solar radiation, turbidity, availability of nutrients, competition, predation, degree of mixing water, and bacterial losses due to sedimentation or death^66^. There was a high prevalence of fecal streptococci in all sampling sites due to their resistance to environmental fluctuations compared to coliform^67^. A significant positive correlation was found between fecal streptococci and NO_2_-N, and NO_3_-N (*r* = 0.79, 0.86, *p* < 0.05), which are related to low water quality. Although these parameters do not have fecal origin, they may be helpful in assessing the water quality from fecal pollution^68^.

**Table 2:** A comparison of the present levels of water quality parameters in Wadi Mariout basins with other Egyptian lakes.

Lake	Wadi Mariout		Mariout		Manzala		Burullus				Edku	
	Eastern	Western										
pH	7.88–7.94	8.01–8.07	7.22–8.68	–	7.56–8.93	8.2–8.7	8–8.8	7.56–9.01	7.32–9.63	7.55–8.95	–	8.2–9
Salinity (‰)	15.37–16	12.28–24.6	2.78–9.68	–	1.17–21.9	–	–	2.91–20.03	–	1.44–34.10	–	4.56–20.6
Trans (cm)	90–115	80–130	5–60	–	10—67	22.5–60	30–45	15–120	–	15–70	–	50–150
DO (mg/l)	4.64–5.15	5.74–7.39	0.4–9	–	0–14.7	3.2–8	6–12.7	4.58–15.50	0.9–19.87	2.10–13.40	–	3.7–15.7
NH_4_^+^ (µg/l)	334.28–577.97	281.78–996.39	120–11,190	–	100–14,620	2600–12,500	1700–5700	128.99–2354.3	13–4450	115.6–11,350	–	88.6–730.6
NO_2_^−^ (µg/l)	6.62–9.72	15.01–51.51	20–360	–	19.74–273.96	1200–6100	600–4000	15.64–447.81	2.98–419.71	14.3–247.9	–	77.3–301.5
NO_3_^−^ (µg/l)	14.94–31.23	92.58–1048.16	80–1990	–	63.75–850.36	1900–4800	1200–3200	33.08–1314.7	130–1640	36.3–1241.1	–	Nd–1241.35
PO_4_^3−^ (µg/l)	66.3–93.05	42.92–69.49	22.67–1317	–	9.9–143.00	2300–8800	1000–2900	56.07–1512.1	–	–	–	43.8–260.6
TP(µg/l)	142.49–172.25	141.07–157.37	66.25–2171	–	–	–	–	–	–	24.6–796.2	–	
SiO_4_^−^ (µg/l)	7.89–11.55	5.89–11.46	351,010,890	–	3.2–10.91	–	–	5.91–29.23	–	–	–	209.7–3655.7
TC (MPN/100 ml)	0–12	4–12	14.4 × 10^4^*	0.18–12.67 × 10^5^	21–240 × 10^4^	7.2 × 10^3^*	1.3 × 10^3^*	7–1100	–	–	0.40–10.77 × 10^4^	6.8 × 10^2^*
FC (MPN/100 ml)	0–3	0–3	10 × 10^4^*	0.04–28 × 10^3^	15–210 × 10^4^	5.2 × 10^3^*	5.4 × 10^2^*	3–340	–	–	ND	4.0 × 10^2^*
FS (MPN/100 ml)	0.24–1.1 × 10^3^	1.1–120 × 10^3^	36 × 10^3^*	ND	0.09–16 × 10^3^	2.4 × 10^3^*	3.9 × 10^2^*	0.44–110 × 10^3^	–	–	ND	1.9 × 10^2^*
TBC at 37 C (cfu/ml)	4–40 × 10^4^	3.2–4.2 × 10^4^	–	1.8–312 × 10^4^	0.2–260 × 10^4^	–	–	0.49–134 × 10^3^	–	–	21–632 × 10^4^	–
TBC at 22 C (cfu/ml)	3.1–35 × 10^4^	27–330 × 10^4^	–	ND	0.4–280 × 10^4^	–	–	0.12–18 × 10^4^	–	–	ND	–
Sample size	15	15	12, 16	9	11	15, 16	15, 16	12	12	12	6	8
Reference	Present study		El-Degwy^51^ and Morsy et al.^62^	Farouk et al.^63^	Mahmoud et al.^52^	Zaghloul et al.^42^ and Morsy et al.^62^	Zaghloul et al.^44^ and Morsy et al.^64^	Al-afify et al.^43^	Alprol et al.^42^	Elsayed et al.^47^	Farouk et al.^64^	Radwan et al.^49^

Table 2 presents a comparison of bacterial load in Wadi Mariout water with the previous studies in some Egyptian northern lakes. Total bacteria in some sites of Egyptian northern lakes exceeded that in Wadi Mariout ponds, which recorded 1.8–312 × 10^4^, 0.2–260 × 10^4^, 0.49–134 × 10^3^, and 21–632 × 10^4^ cfu mL^−1^ in Mariout, Manzala, Burullus, and Edku lakes, respectively. Total and fecal coliform values were very rare in Wadi Mariout water compared with that in some of Egypt’s northern lakes, which reached 10^5^ in Mariout, Manzala, and Edku lakes, while in Burullus Lake ranged from 7 to 1100 and from 3 to 340 for total and fecal coliforms, respectively. Although fecal streptococci were enriched in all sites of Wadi Mariout water, Egypt’s northern lakes showed levels exceeding these by one to two times^5,52,63,64^. The bacterial pollution indicators in the Wadi Mariout water have been within the permissible values of the Egyptian Standard Ministry of Health^69^, and the European Commission Manual^70^. Where they accept the guide values for total and fecal coliform of seawater up to 50 cfu ml^−1^ and 1 cfu ml^−1^, respectively, this finding is different from that reported of Mariout Lake by Farouk et al.^63^. According to Egyptian Law (48/1982) for the protection of the River Nile and waterways from pollution, allowable limits are 100 cell/100 ml for total and fecal coliforms, and 1000 cells/100 ml for fecal streptococci. Wadi Mariout water was within the permissible values for total and fecal coliforms, but S10 exceeded allowable limits for fecal streptococci. Northern lakes recorded very high pollution levels compared with the Wadi Mariout and the Egyptian Law allowable limits in Table 2. Morsy et al.^62^ reported that the levels of total coliform, fecal coliform, and fecal streptococci bacteria were high for the four lakes (Mariout, Manzala, Burullus, and Edku Lakes), indicating the presence of pathogens in the water. Despite Hassan and Badran’s assertion that Wadi Mariout ponds are free from direct industrial or domestic pollution sources^45^, our results (as at station 1) suggest otherwise, indicating a potential contamination by a mix of industrial or sewage wastewater within the agricultural drainage that feeds the lakes.

#### Phytoplankton dynamics

**Fig. 3:**
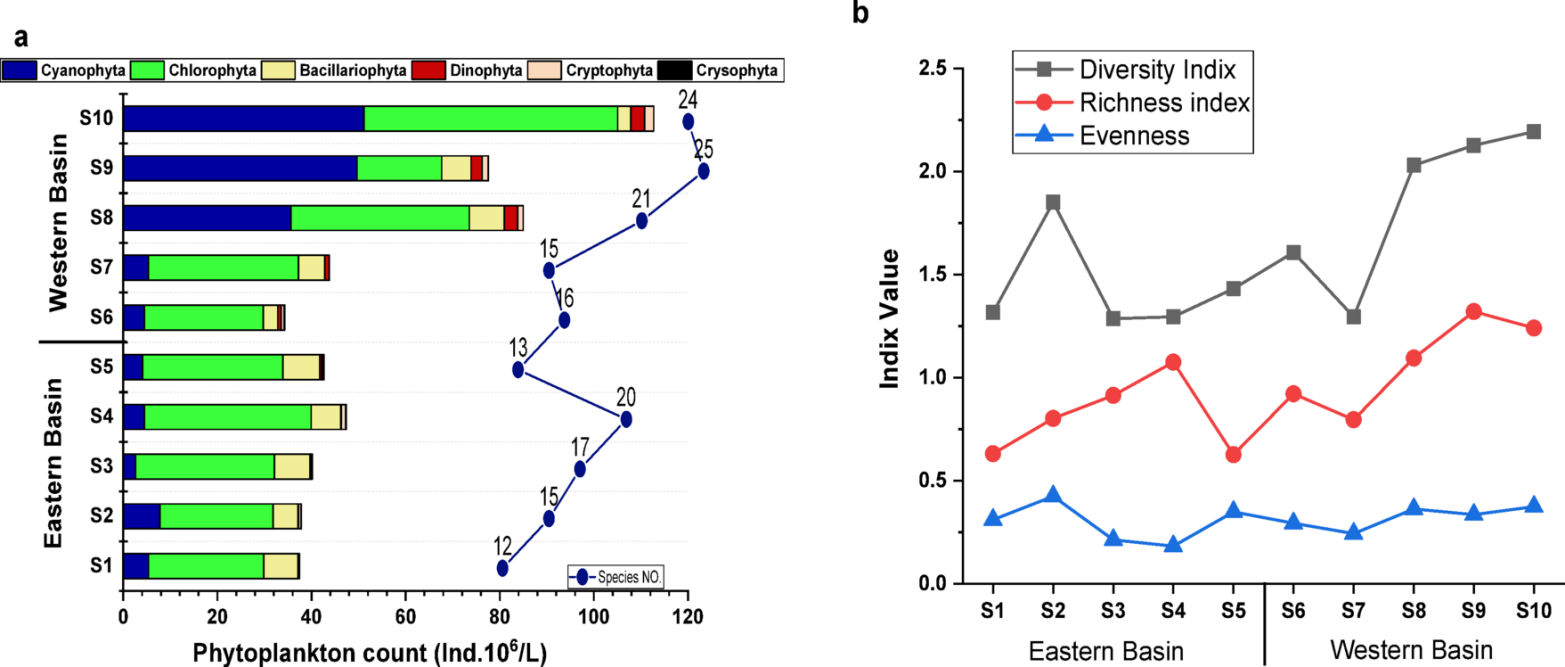
(**a**) Variations in the density of phytoplankton groups (Ind.10^6^.l^−1^) and species richness, (**b**) Diversity indices of phytoplankton community at the eastern and western basins of Wadi Mariout Pond.

The diversity of phytoplankton species from different algal groups is a good indicator of water quality, and small ponds can support a more diverse phytoplankton community than a large one^71,72^. In the present study, the species diversity in Wadi Mariout is low (37 spp.) (Table S3, in the supplementary file) compared to the Egyptian Mediterranean Lakes: Manzala (383 spp.), Mariout (376 spp.), Bardawil (333 spp.), Burullus (247 spp.), and Edku (183 spp.)^73^ and the Eastern Poland lakes (Czarne, Glinki, and Gumienek) (178 spp.)^74^. The low number of species may be attributed to the age and size of the pond, which could influence the water characteristics and the phytoplankton diversity and abundance^75^. The identified species belong to Chlorophyta (12 spp.), Cyanophyta (10 spp.), Bacillariophyta (8 spp.), Dinophyta (4 spp.), Cryptophyta (2 spp.), and Crysophyta (1 sp.). The phytoplankton densities and species richness varied significantly between the two basins (Fig. 3a). The eastern basin fluctuated within a narrow range of phytoplankton density (37.4–47.4 × 10^6^ Ind.l^−1^) with an average of 41.1 × 10^6^ Ind.l^−1^ and (12–20 species). Meanwhile, the western one was more flourished by the phytoplankton community (34.3–112.7 × 10^6^ Ind.l^−1^) and the species number varied between 15 and 25 species (Fig. 3a). The phytoplankton community was dominated by green microalgae (24–35.4 × 10^6^ Ind.l^−1^; 63–75%) and (18–53.9 × 10^6^ Ind.l^−1^; 23–74%) in the eastern and western basins, respectively. The second dominant group was cyanobacteria (2.6–7.8 × 10^6^ Ind.l^−1^; 6.5–20.6%) and (4.5–51 × 10^6^ Ind.l^−1^; 12–64%) (Fig. 3a). The other groups contributed to the total community in the Wadi Mariout ponds as follows: diatoms (2.5–19.5%), dinophyta (0–3.3%) and cryptophyta (0.22–2%). The dominant green species were *Botryococcus* sp, and *Chlorella sorokiniana* Shihira & R.W. Krauss 1965, comprising (20–73%) of the total abundance. While the dominant cyanobacteria species were *Chroococcus dispersus* (Keissler) L. 1904, *Chroococcus dispersus* var. *minor* G.M. Smith 1920, *Chroococcus minimus* (Keissler) L. 1904, *Merismopedia glauca* (Ehrenberg) Kützing 1845, and *Synechococcus* sp with a share percent to total phytoplankton abundance (6–56%). The diatoms were dominated by *Cyclotella ocellata* Pantocsek 1901, *Thalassiosira* sp, and *Detonula* sp comprising (2.4–19.5%), and the dinophyta was dominated by *Gyrodinium* sp and *Prorocentrum donghaiense* D. Lu 2001 with a shared percentage of 0–3.3%. The abundance of Cyanophyta and Chlorophyta species is similar to that in Mariout Lake, attributed to the high concentration of nutrient salts (N: P ratio, 14.9–25.7) through wastewater supplies entering the lake^76^. The chlorophyta plays a role in transferring energy to zooplankton^75^. The highest phytoplankton abundance in the two basins (Fig. 3a), Chl-*a* (7 < Chl-*a* ≤ 30 µg/L), and species richness may be attributed to eutrophication, which increases the density during summer, smiliar to Edku Lake^73^. Water eutrophication causes an increase in phytoplankton densities, thereby affecting zooplankton predation, resulting in ecological instability and decreased biodiversity^77^. This is confirmed by the low Shannon’s diversity values ranging from 1.29 to 1.3 (S1, S3, S4, and S7) to 2.0-2.11-2.2 (S8, S9, and S10) (Fig. 3b). Also, the species richness of Margalef (0.63–1.3) and the evenness values (0.18–0.42) (Fig. 3b) indicate that the phytoplankton community is dominated by a few species of numbers (i.e. an uneven distribution of species)^78–82^, suggesting a degraded and ecologically unstable environment.

#### Biochemical contents and bio-polymeric particulate organic carbon of phytoplankton

**Fig. 4:**
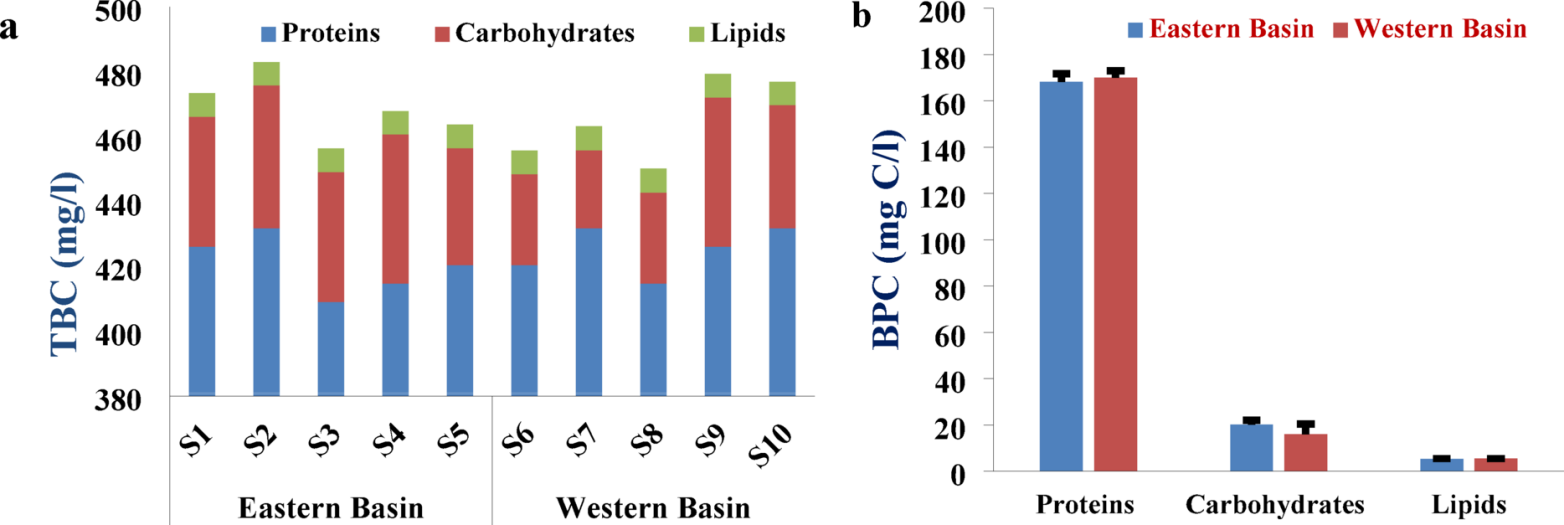
(**a**) Total biochemical content (TBC) of phytoplankton, (**b**) Bio-polymeric organic carbon (BPC) based on carbon sequestration at the eastern and western basins of Wadi Mariout Pond.

The selected sites along Wadi Mariout Pond showed a convergence of results for the three studied biochemical parameters, including proteins (422.73 mg/l, on average), carbohydrates (37 mg/l, on average), and lipids (7.22 mg/l, on average), where the lowest value of photoprotein was observed at S3 (409.1 mg/l) (Fig. 4a). This result was confirmed by the rapprochement of the percent of the abundance of the two dominant classes of phytoplankton cells, Cyanobacteria, and Chlorophyta at all the pond sites (76.3–86.2%). Phytoplankton proteins represent the major biochemical parameter, followed by carbohydrates and then lipids. These results agree with the phytochemical contents at Burullus Lagoon, where phytoplankton organic matter is dominated by proteins^83^. Protein represented the largest pool of cellular C and N in algal species under steady-state growth^84^. A relatively elevated nitrogen or protein content in the algal biomass of the Salton Sea in the Imperial Valley of California was observed, likely due to the higher total nitrogen concentration in the source water^85^. Comparable findings have been documented for biomass exposed to excessive nitrogen inputs, which can be linked to increased protein content associated with photosystem biosynthesis^86^. The predominance of phytoproteins revealed that the phytoplankton of the pond is physiologically healthy with high relative growth rates, and high protein content suggests that phytoplankton had no nitrogen depletion in the water body^87^. Algae have a high energy value and are a source of biologically active substances, proteins, fats, and carbohydrates^88^.

Phytoplankton’s carbon metabolism strategy affects their carbon fixation potential, where phytoplankton fixes carbon into organic forms to produce storage intracellular proteins, carbohydrates, and lipids^89^. Particulate bio-polymeric organic carbon (BPC) of phytoplankton along Wadi Mariout Pond (192.64 mg C/l) was associated with the high extent bio-polymeric C of proteins µ = 87.8%, indicating that carbon was highly incorporated into cellular proteins (169.1 mg C/l) more than carbohydrates and lipids (18.13 and 5.42 mg C/l for carbohydrates and lipids, respectively) (Fig. 4b). During the study of the hot season, CO_2_ absorption in the water was higher than in the cool season, as indicated by Phrommarat and Buawech^90^.

When the light was sufficient and the temperature suitable, algal activity remained high, and fixed carbon flowed to the protein needed to synthesize new individuals^91^. According to the spatial variations, S2 represents the optimum nutritional value (482.92 mg/l) along the studied pond, as illustrated in Fig. S17 (in the supplementary data). Where green microalga *Botryococcus* sp. detected in the same period of study represents the maximum number (23.7 cells × 10^4^ l^−1^). *Botryococcus* sp. contains 22% protein content and hydrocarbons up to 75% of dry weight^92^.

According to the spatial variations between the eastern and western ponds, there was a rapprochement in the biochemical contents, where the eastern basin represents the maximum concentration (468.85 mg/l), Fig. 3b, where biopolymeric particulate organic carbon of phytoplankton reached 193.77 mg C/l. Habitat conditions can alter the carbon metabolic strategy of algae, affecting the biochemical composition of the algae, which is effective in sequestering carbon in organic components^93^. This result agrees with the increase in the average value of PO_4_^3−^ at the eastern basin (76.8 µg/l) compared to the western one (55.3 µg/l). Phosphorus was considered the primary limiting factor to algal growth, which is an indicator to evaluate the quality and level of water fertility^94,95^. The findings indicate that the metabolism of phytoplankton cells can be significantly affected by abiotic environmental factors due to their ability to adapt. The high proteins: carbohydrates ratio (> 11) noted in the biochemical composition reflects a high level of metabolic activity under nitrogen-rich conditions. When the phytoplankton are in a suitable physiological state and have sufficient nutrients, they can support the exponential growth phase, which is characterized by a high carbohydrate-lipid ratio (> 5). The metabolism of algae is greatly impacted by environmental changes^96^.

#### Zooplankton communities

**Fig. 5:**
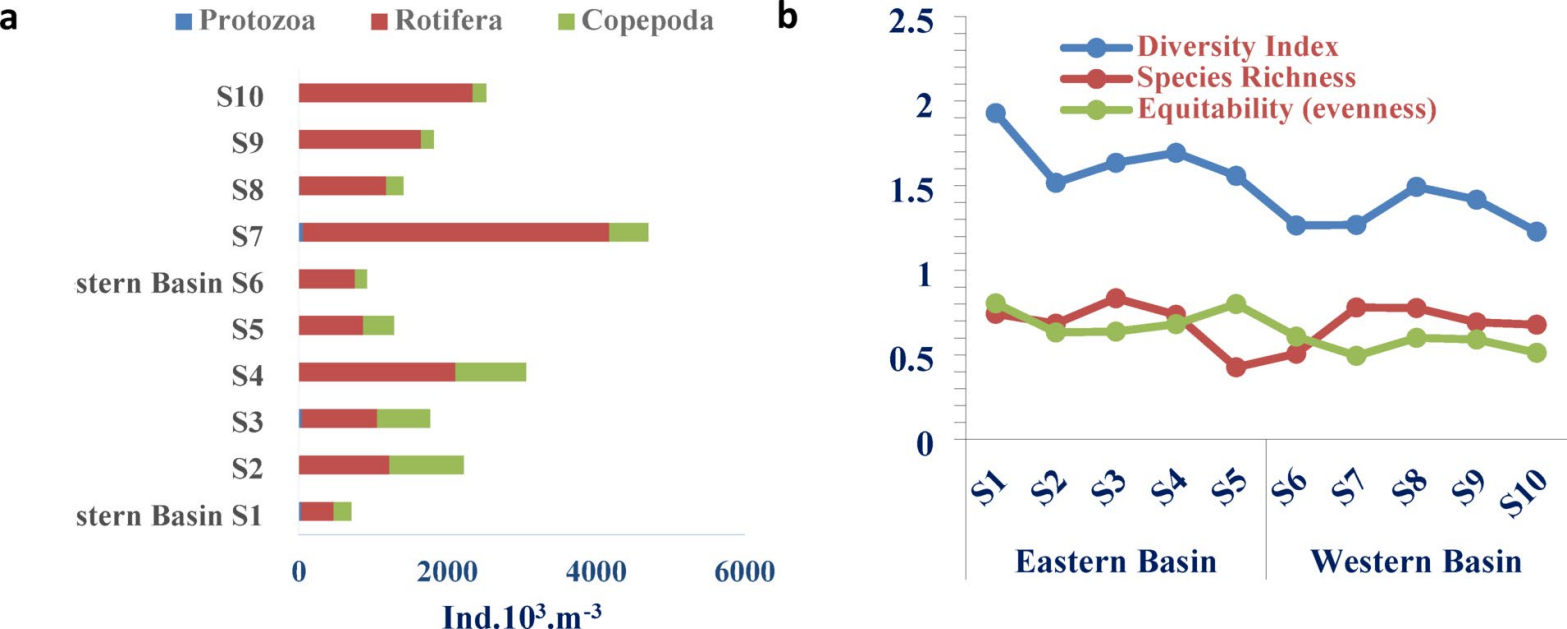
(**a**) Variations in the numbers of zooplankton and their groups (Ind.10^3^ m^−3^) and (**b**) Diversity indices of zooplankton community at the eastern and western basins of Wadi Mariout Pond.

The average of zooplankton detected in the study area was 1,804,200 Ind./m^3^ in the eastern basin and increased to 2,323,200 Ind./m^3^ in the western basin of Wadi Mariout Pond. Due to the outstanding peak of 4,806,000 Ind./m^3^ at sampling S7, site 1 showed the lowest zooplankton count of 705,000 Ind./m^3^ (Fig. 5a). Zooplankton communities in the studied area are composed of Rotifera and Copepoda, in addition to some members of Protozoa (0.8% and 0.6% of total zooplankton in the two basins) and an odd species of Cladocera only detected in sampling S7. Regarding population density, Rotifera was the most dominant group, forming about 62.5% and 86.3% of total zooplankton in the eastern and western basins, respectively. Rotifera being the most important zooplankton group in the four delta lakes (Mariout, Edku, Burullus, and Manzala), its density ranged from less than 80% to more than 90% of the total zooplankton population in the lakes^97^. The dominance of Rotifera refers to the fact that these organisms are opportunists or *r* strategists, characterized by their small size, short life cycles, and great tolerance to different environmental factors^98^. Small-sized zooplankton groups like Rotifera are known to be indicators of eutrophication^26^. Rotifers are growing significant populations in eutrophic conditions due to their effective reproduction ability and brief life cycles^99^. In Lake Edku, the zooplankton community changed from Cladocera’s dominance to rotifers and small zooplankton owing to eutrophication, decreased heterogeneity of its ecosystem, and an increase in organic pollution^100,101^.

Thirteen rotifer species were counted with an overall average of 1,128,000 and 2,005,200 Ind./m^3^ in the eastern and western basins, respectively. The rotifer genus *Brachionus* dominated the other genera; it is represented in the studied area by seven species: *B. urceolaris*,* B. plicatilis*,* B. quadridentatus*,* B. angularis*,* B. calyciflorus*, *B. leydigii*, and *B. rubens*. This genus formed about 77% of the total rotifer population in each of the two studied basins. *Brachionus urceolaris* (about 60% and 69% of total rotifers in the eastern and western basins, respectively) and *B. plicatilis* (12.4% and 4.4% of total rotifers) were the dominant species of the genus.

*Hexarthra mira* followed *Brachionus urceolaris* in abundance and formed about 18% and 20% of total rotifers in the eastern and western basins of Wadi Mariout, respectively. Zakaria et al.^102^ investigated the ecological status of zooplankton in Mariout Lake over seven successive years, with rotifers being the abundant zooplankton group and *B. urceolaris* and *B. calyciflorus* being the most abundant species at all basins over the study period. The dominance of *Brachionus* spp. indicated the polluted nature of Lake Mariout^102^ and its occurrence is an indicator of aquatic ecosystem status^101^. The presence of *Brachionus* species is an indicator of the eutrophic nature of water bodies^103,104^. Mageed^103^ also observed that Lake Manzala was transformed from a marine ecosystem to a eutrophic, nearly freshwater system with the domination of Rotifera taxa (about 97%). Eutrophication led to degraded biota in which the species richness decreased over time due to high oxygen consumption by increased phytoplankton and aquatic vegetation^105^. Some rotifers detected in the present study were significantly influenced by the PO_4_ concentrations (*Brachionus* (*p* = 0.03), *Polyarthra vulgaris* (*p* = 0.04), and *Asplankhna sieboldi* (*p* = 0.03)) and considered as indicators of eutrophic conditions. Attayde and Bozelli^106^ demonstrated that *Brachionus*,* Asplanchna*,* Filinia*, and *Polyarthra* were good indicators of the eutrophic environment.

Copepoda, represented by its young forms (nauplii and copepodites), contributed about 36.7% and 13.0% of the total zooplankton count in the eastern and western basins, respectively. The density values of Copepoda significantly correlate with salinity (*r* = 0.66, *p* = 0.03). It is common for immature forms, especially nauplii, to be more abundant in freshwater. These forms occupy different trophic niches than adults; nauplii and copepodites that feed by filtering food are herbivores, while adults that eat by capturing food are carnivores^98^. Edmondson^107^ referred to the abundance of larval forms of Copepoda due to their continuous reproduction in tropical regions.

The diversity indices predict changes in a precise zooplankton community structure. According to El-Sebaie et al.^108^ and Olivera et al.^109^, these indices are typically employed to evaluate the ecological diversity within and/or between specific aquatic environments. S3 and S7 had the highest values of species richness (0.834 and 0.780) and the greatest number of species (13), according to the zooplankton community’s diversity indices in Wadi Mariout Pond. The Shannon–Wiener index of zooplankton diversity varied from H′ = 1.228 (S 10) to 1.929 (S 1). The highest evenness (equitability) was recorded at S1 and S5 of the eastern basin, and the minimum value was detected in S7 (Fig. 5b). In a study of the zooplankton community in the Niger Delta, Nigeria, using different diversity indices, the Shannon–Wiener index ranged between 2.611 and 3.571 revealing moderate pollution in most of the sampling sites. Moreover, evenness ranged from a minimum value of 0.576 to a maximum of 0.864, which reflects the effect of anthropogenic activities^110^.

### Sediments

#### Grain size analysis

From the late Pleistocene to the Holocene, there is a broad stratigraphic succession found in the river delta and Mediterranean delta regions. As for the Nile Delta, it is divided into three main stratigraphic sequences. The first is the late Pleistocene, which is fluvial deposits; above that is the early Pleistocene layer, which is shallow sandy marine deposits. The last layer, which is considered the most important layer, is the Holocene, where the delta and the northern lakes were formed. This resulted from the change in sea level. The sea cleared and some hills separated the lakes and shallow areas, forming lakes^111^. The composition of Wadi Mariout basins was gravel (fraction > 1 mm granules and shills), sand varying between fraction 1 mm and 63 μm, and mud less than 63 μm (Table 3). In the eastern basin, sedimentation processes are increasing, indicating a calmer environment and more mud. In the western basin, where there is an increase in sand, there is also an increase in erosion due to an active environment. According to Folk’s classification system, the sediment types in the two basins are gravelly muddy sand, sandy mud (Table 3). The results of comparing the basins of Wadi Mariout with the northern Delta lakes (Idku, Manzala, and Burullu) showed that the two basins of Wadi Mariout have sedimentation environments that are comparable to those of Idku and Manzala lakes. In terms of water content and organic matter in sediment, the two basins of Wadi Mariout, Edku, and Manzala are similar to each other, despite being distinct from Lake Burullus^112^.

#### Bacteriological analysis

**Fig. 6:**
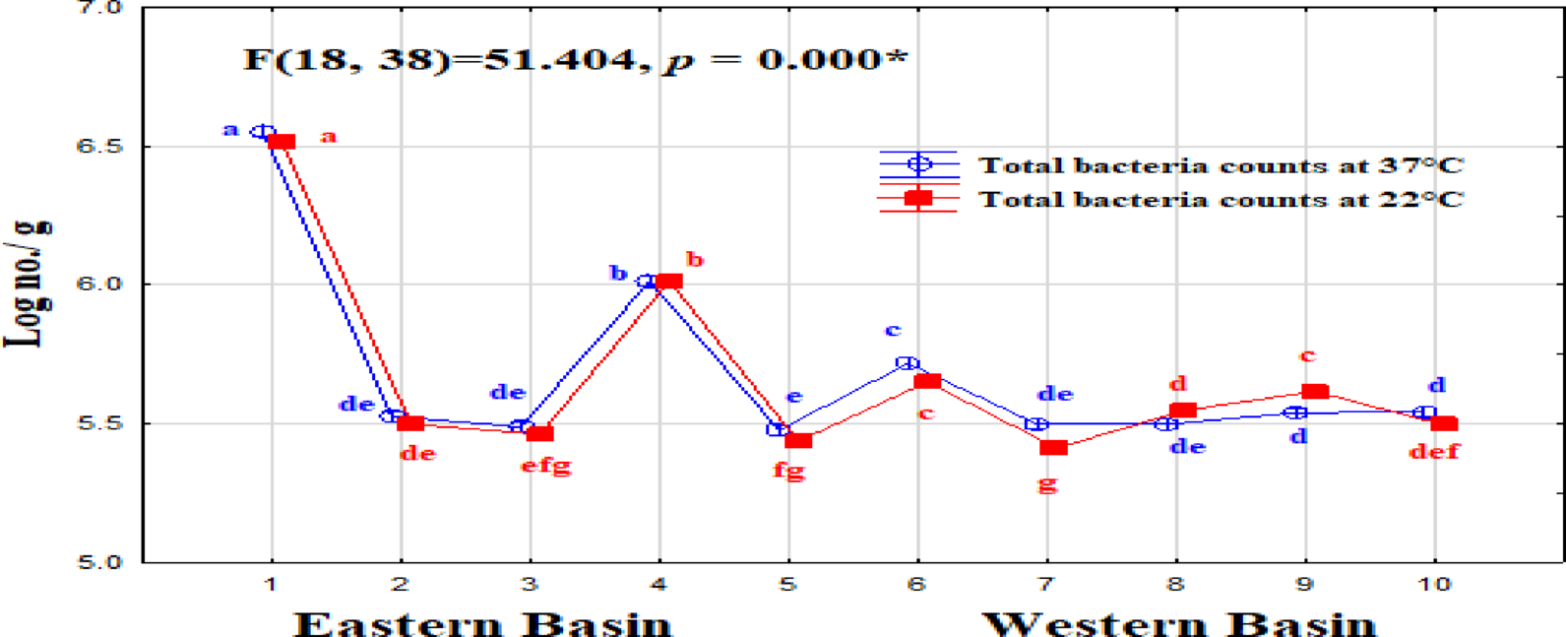
Spatial changes in a microbial load along Wadi Mariout sediment (ANOVA analysis).

As shown in Fig. 6, the analysis of variance (ANOVA) indicated significant differences in total bacterial counts (TBC) developed at either 22 and 37 °C. The maximum value of total bacterial counts at 37 and 22 °C was found in S4 (up to 105 cfug^−1^), and the minimum value was in western basin sites. Total coliforms ranged from 15 to 3384 cfug^−1^, fecal coliform ranged from 0 to 150 cfug^−1^ and fecal streptococci showed enrichment values in all samples ranging from 2.4 × 10^2^ to 12.0 × 10^4^ cfug^−1^. Among sites, S10 recorded the highest values of FS in sediment (Table S4). Sediment samples show a higher bacterial load than water samples. Sediments also can impact the quality of water. Sediments can contain 100 to 1000 indicator bacteria than the overlying water; they can serve as reservoirs for fecal pollution. Most coliform bacteria in aquatic systems are associated with sediments, and these relations influence their survival and transport characteristics^63,113^. Farouk et al.^63^ reported that total coliforms ranged from 0.30 to 82.00 × 10^4^ cfug^−1^ and fecal coliform ranged from 0.16 to 85.00 × 10^3^ cfug^−1^ in Mariout Lake, which indicates higher pollution than in Wadi Mariout. While total bacterial counts varied from 0.32 to 4.0 × 10^5^.

**Table 3:** Sediment fractions, water content, and organic matter at the Eastern and Western basins of Wadi Mariout pond.

	Site	Gravel	Sand	Mud		Feature	Water content %	OM %
				Silt	Clay			
Eastern Basin	S1	13.58	39.8	24.9	21.7	Gravelly Mud	55.65	14.88
	S2	4.1	44.9	27.7	23.2	Sandy Mud	52.38	12.33
	S3	7.19	41.6	27.5	23.6	Gravelly Mud	61.24	18.19
	S4	5.23	42.1	26.9	25.8	Gravelly Mud	74.97	12.9
	S5	5.42	53.4	25.2	16	Gravelly Muddy Sand	49.52	13.39
Western Basin	S6	9.82	43.9	26.3	19.9	Gravelly Mud	60.21	14.98
	S7	7.96	50.7	25.6	15.7	Gravelly Muddy Sand	48.34	12.31
	S8	6.19	59.8	22.7	11.3	Gravelly Muddy Sand	63.4	15.65
	S9	1.15	44	34.2	20.6	Sandy Mud	56.18	12.62
	S10	6.04	50.7	23	20.2	Gravelly Muddy Sand	49.66	10.68

#### Mactrobenthos

A total of 10 species belonging to three phyla of macrobenthic fauna were observed at Wadi Mariout Pond (Table S3). This result partially agrees with Khalil and Koussa^114^, who recorded 14 species, belonging to only two major groups: Mollusca (13 species) and Arthropoda (Insecta: one species) in Lake Mariout. Ten species were recorded in the eastern basin, while five species were recorded in the western basin. The main types were the sedentary Polychaeta, Bivalvia, and insects. Overall, a total of 9 families, including 37,034 individuals, were recorded in sediment samples from Wadi Mariout ponds; of these, 4 arthropods, 3 annelids, and 3 molluscs. Mollusca were the dominant group in two basins at Wadi Mariout pond (Table S3). The rare representation of macrobenthic species could be attributed to the nature of the bottom; El-Shabrawy and Gohar^115^ recorded that the nature of the bottom has a selective influence on the quality of benthos, and also, salinity was the main factor responsible for changes in faunal composition. Ramadani et al.^116^ and Mitwally^117^ reported poor benthic fauna (meiofauna and macrofauna) in the Northern Egyptian Lake sediments. Due to the poor distribution of macrobenthos, the Wadi Mariout Pond may differ somewhat from some northern lakes such as Mariout, Manzala, and Burullus lakes, where Khalil et al.^118^ listed about 50 species in Bardawil Lake and noted a notable decline over the summer In Burullus Lake, Abdel Gawad and Abdo^119^ identified 27 species belonging to twenty-three families within eight classes and three phyla: Mollusca, Annelida, and Arthropoda. Mollusca recorded the highest number of species (19 species), while Annelida, and Arthropoda recorded 4 species. In Manzala Lake, there are fifteen species belonging to the three groups Mollusca, Annelida, and Arthropoda, and the average population density of total macrobenthic invertebrates showed its maximum value in winter and autumn, while its minimum value was recorded in summer^120^.

**Fig. 7:**
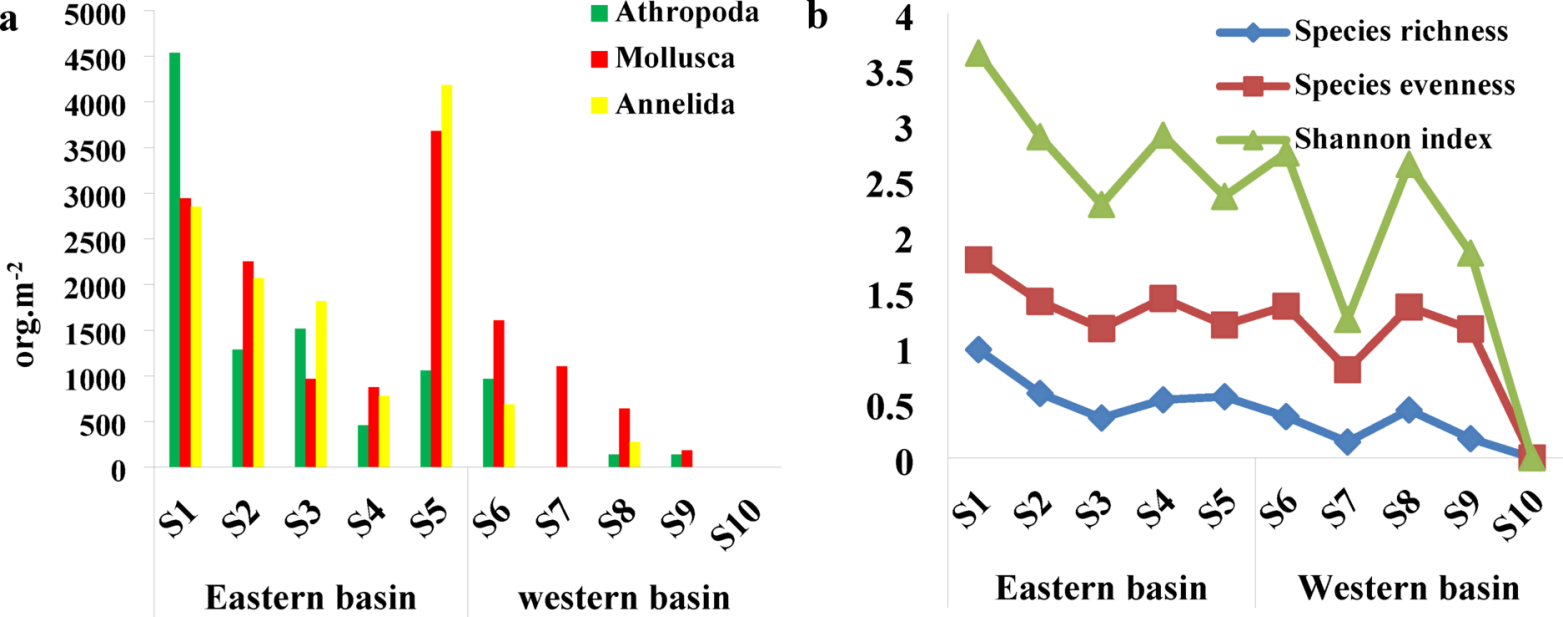
(**a**) Population density of macrobenthic invertebrate groups in the community and (**b**) Diversity index, species richness and species evenness at the eastern and western basins of Wadi Mariout Pond.

The total community composition of macrobenthic invertebrates (MBI) during the investigated study was 31,284 org. m^−2^ and 5750 org. m^−2^ for the eastern and western basins, respectively. Mollusca (62% to MBI count) was the dominant group by three taxa (*Venerupsis aurea*,* Cerastoderma glaucum*, and *Mytilus* sp.) at the western basin in Wadi Mariout Ponds, for the eastern basin; Annelida was the dominant group (38% to MBI count) (Fig. 7a). The main sites harbored the highest density of macrobenthos, with a maximum value of 10,331 org.m^−2^ at S1 and 3266 org.m^−2^ at S6 in the eastern and western basins, respectively. This high population density was due to a large number of Chironomidae larvae and Tubificidae sp. (most tolerant groups), while a sharp decline in total MBI was noticed at S 4 in the eastern basin that counted 2116 org.m^−2^, while MBI was completely absent in the western basin at S 5. The biodiversity of macrobenthos revealed that S1 in the eastern basin recorded the highest species richness (10 species). The present study represented *Cerastoderma glaucum*, the most dominant species of macrobenthic invertebrates in Wadi Mariout Ponds. The annelid Tubificidae sp. were the most dominant annelid genera in two basins (34.8%, and 16.8% of total MBI count in the eastern and western basins, respectively); they were recorded in the most studied sample site. The arthropod community consisted primarily of chironomids, which accounted for 24.6% and 21.6% of the total MBI count in the eastern and western basins, respectively. These findings suggest a potential correlation between chironomid abundance and sediment conditions. The prevalence of chironomids signifies their wide-ranging habitat preferences and their capacity to adjust to seasonal fluctuations. The sediment at all sites consisted predominantly of a mixture of empty shells, shell pieces, and calcareous tubes from *Ficopomatus enigmaticus*.

### Macrobenthic groups

#### Mollusca group

Mollusca species, which were collected from Wadi Mariout, showed the highest value of 3680 org.m^−2^ in S5 at the eastern basin and S6 (1610 org.m^−2^) in the western basin. This result is mainly attributed to the dominance of *Cerastoderma glaucum*; it formed about 24.6% and 40% of total MBI in the eastern basin and western basins, respectively. Also, these results may be related to elevated ammonia concentrations in these sites at the two basins. The obtained results are in confidence to Khalil and Koussa^114^. The lowest values were recorded in S4 and S9 for the eastern and western basins, respectively, and the complete absence of this group was recorded in S10 at the western basin (Fig. 7a).

#### Arthropoda group

Arthropoda is recorded in S1 in the eastern basin and S6 in the western basin at Wadi Mariout. The highest value is mainly attributed to the dominance of Chironomidae larvae. This result was attributed to the ability of Diptera species to exhibit certain adaptations to survive in severely polluted conditions water^121,122^ while the lowest value was recorded in S 4 for the two basins, and the complete absence of this group was recorded in S 4 and 8 (Fig. 7a).

#### Annelida group

The highest standing crop of this group is at S 5, with a value (of 4186 org.m^2^) in the eastern basin and S6 in the western basin, which is mainly attributed to the dominance of Tubificidae sp., while the lowest values were recorded in S4 for the eastern basin and S8 for the western one (Fig. 7a).

The presence of diverse species in an aquatic environment plays a crucial role in performing many tasks that are essential for the ecosystem. Consequently, the reduction in the number of species in the studied habitats is regarded as a decline in biodiversity in contaminated ecosystems, resulting in a loss of functional biodiversity^123^. According to Brandt and Ebbe^124^, the reduction in biodiversity results in a decline in ecosystem function, which in turn destroys habitats. The primary cause of this is the pollution resulting from the actions of the human population^125^. In addition, Ramadan et al.^126^ and Hassan^127^ ascribed the decline in biodiversity to escalated water pollution. The present study revealed that the biodiversity of macrobenthos Wadi Mariout Pond at S1 recorded the highest species richness and Shannon–Weaver index value (d = 0.9737 and H′ = 1.858). It could be attributed exclusively to the dominance of Chironomidae larvae. On the contrary, S 7 recorded the least species richness and Shannon Weaver index value (d = 0.1427 and H′ = 0.4506) (Fig. 7b).

### Statistical analysis

#### Canonical correspondence analysis (CCA)

**Fig. 8:**
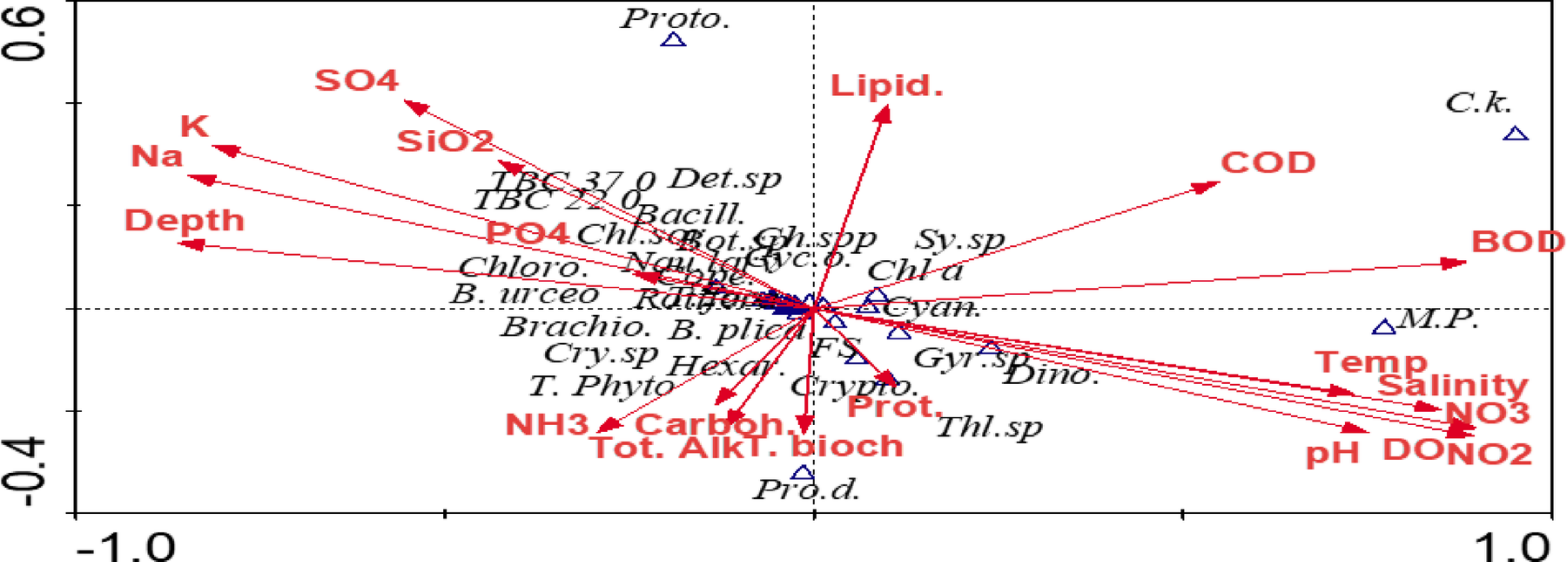
Canonical correspondence analysis (CCA) ordination showing the correlations between zooplankton, phytoplankton community, and bacteria with selected environmental variables and total biochemical content in Wadi Mariout water. (Temp.) Temperature, (Brachio.) *Brachionus*, (B. urceo.) *B. urceolaris*, (B. plicat.) *B. plicatilis*, (Hexar.) *Hexarthra mira*, (Nau. larva) Nauplius larva, (Cope.) Copepoda, (Proto.) Protozoa, (T. zoo.) Total zooplankton, (Chl a) Chlorophyll a, (Ch. spp) *Chroococcus* spp, (C.k.) *Coelosphaerium kuetzingianum*, (M.P.) *Merismopedia punctate*, (Sy. sp) *Synechococcus* sp, (Bot. sp) *Botryococcus* sp., (Chl. sor.) *Chlorella sorokiniana*, (Cyc. o.) *Cyclotella ocellata* (Thl. sp) *Thalassiosira* sp., (Det. sp) *Detonula* sp., (Gyr. sp) *Gyrodinium* sp., (Pro. d.) *Prorocentrum donghaiense*, (Cry. sp) *Cryptomonas* sp, (T. Phyto) Total Phytoplankton, (Cyan.) Cyanophyta, (Chloro.) Chlorophyta, (Bacill.) Bacillariophyta (Dino.) Dinophyta, (Crypto.) Cryptophyta, (TC.) Total coliform, (FC.) Fecal coliform, (FS.) Fecal streptococci, (TBC 37 °C). Total bacteria counts at 37 °C, (TBC 22 °C). Total bacteria counts at 22 °C, (prot.) protein, (carboh.) carbohydrates, (T. bioch) Total biochemical content.

In Water, the CCA clarifies the effect of the physico-chemical and bacteriological variables on the phyto and Zoo-plankton composition and abundance and their correlation with the bacteriological analysis (Fig. 8). The most important factors affecting biota are water temperature, salinity, depth, pH, K, Na, BOD, DO, NO_3_, NO_2_, and NH_4_. The impact of environmental conditions on phytoplankton composition and dominance is evident in many studies^3,128–132^. Talling and Lemoalle^133^ reported that diatoms are increased by high silicate concentration, while nitrate, phosphate, and sulfate affect the abundance of chlorophyta and cyanobacteria. This is evident in the present study, Fig. 8. The cyanobacteria, *Chroococcus* spp, Dinophyta, *Synechococcus* sp, *Thalassiosira* sp, *Merismopedia punctate*, Cryptophyta, *Coelosphaerium kuetzingianum*, and *Gyrodinium* sp. were positively associated with the salinity, temperature, DO, NO_3_, NO_2_, pH, and BOD (Fig. 8). These variables were negatively associated with most taxa of zooplankton, such as *Brachionus* spp., *Hexarthra mira*, *Nauplius larva*,* Copepoda*, and Total zooplankton. Also, these variables were negatively correlated with total bacteria counts at 37 °C, total bacteria counts at 22 °C, and fecal coliform (FC). The green algae (*Botryococcus* sp.) and the Dinophyta species (*Prorocentrum donghaiense*) were greatly affected by COD. Chlorophyll *a*, *Cryptomonas* sp., and Cryptophyta were moderately affected by the physiochemical variables. Meanwhile, Chlorophyta, Bacillariophyta, and their dominant species (*Chlorella sorokiniana* and *Cyclotella ocellata*) were affected by depth, PO_4_^3−^, Na, K, and SiO_4_^−^. The CCA diagram shows the least influential factors on species composition in Wadi Mariout are carbohydrates, protein, total alkalinity and total biochemical content. (Fig. 8).

**Fig. 9:**
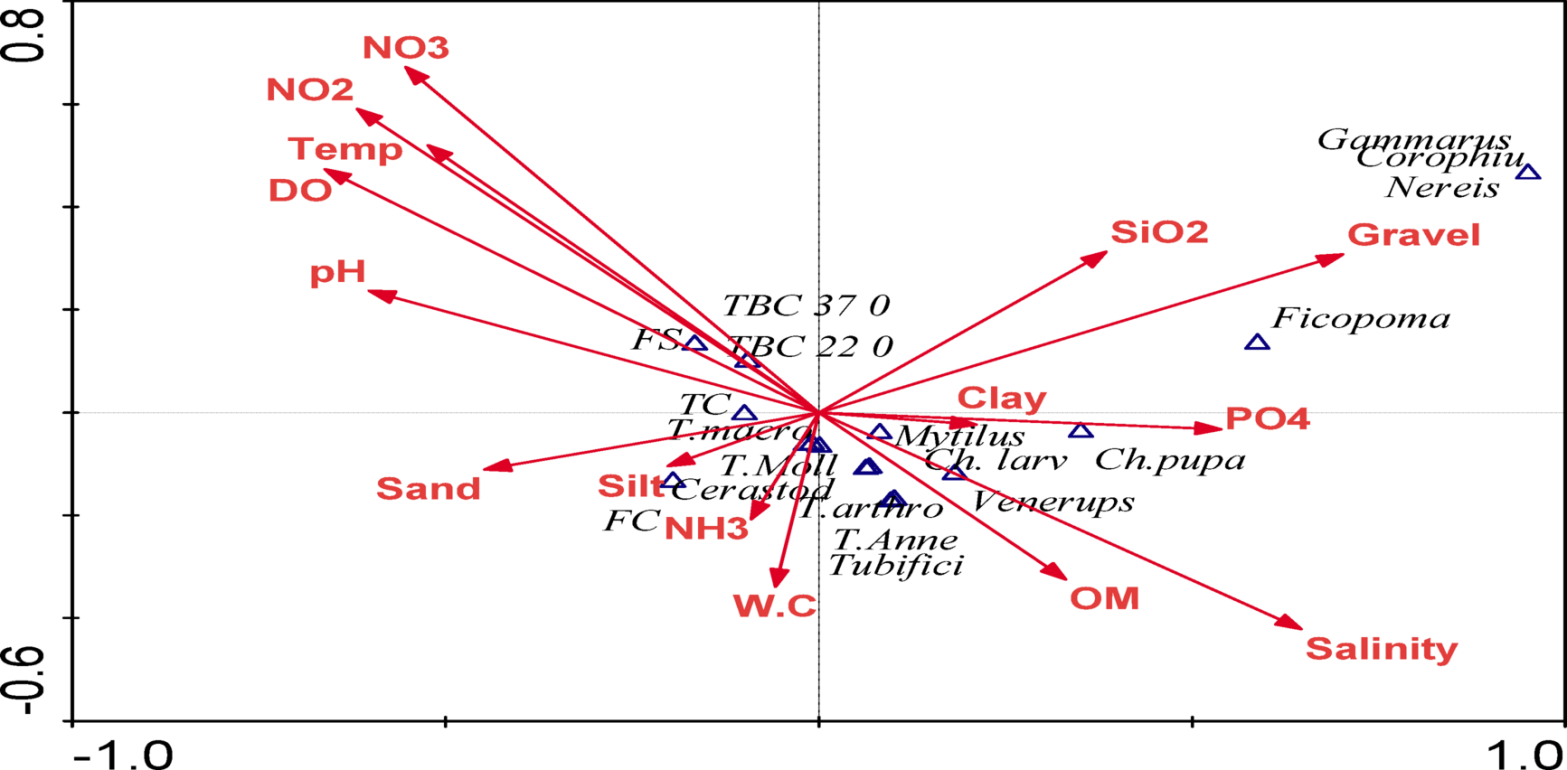
Ordination diagram showing the result of CCA analysis for MBI and bacteria in wadi Mariout sediment (*Ch. larv*,* Chironomid larva; T. arthro*,* total Arthropoda; T. macro*,* total macrobenthos; T. Moll*,* total Mollusca; T. Anne*,* total Annelida; Corophiu.*,* Corophium volutator*,* Ficopoma.*,* Ficopomatus enigmaticus*,* Tubifici.*,* Tubificida.*,* Cerastod.*,* and Cerastoderma glaucum*; Fc., Fecal coliform; Tc, Total coliform, Fs, Fecal streptococci; TBC 37, Total bacteria count at 37 °C; TBC 22, Total bacteria counts total at 22 °C).

In sediment, canonical correspondence analysis (CCA) ordination was performed on the abundance of the collected MBI groups, sediment bacteria, and the corresponding studied environmental variables (Fig. 9). The results of CCA reveal that MBI composition and bacteria were mostly related to salinity, followed by dissolved oxygen, water temperature, pH, and NO_2_. Salinity, organic matter, and water content showed a positive association with the abundance of a total of each MBI, Arthropoda, Annelida, and Mollusca and a negative association with most of the studied environmental variables such as water temperature, pH, and NO_2_, and DO. In contrast, *Gammarus aequicauda*, *Corophium volutator*, *Ficopomatus enigmaticus*, and *Nereis diversicolor* are most correlated to SiO_2_ and gravel. The CCA biplot diagram has shown that bacteria count at 22 °C, bacteria count at 37 °C, Fecal streptococci, and total coliform have a negative correlation with salinity while positively correlated with water pH, dissolved oxygen, and water temperature (Fig. 9).

#### Cluster analysis

**Fig. 10:**
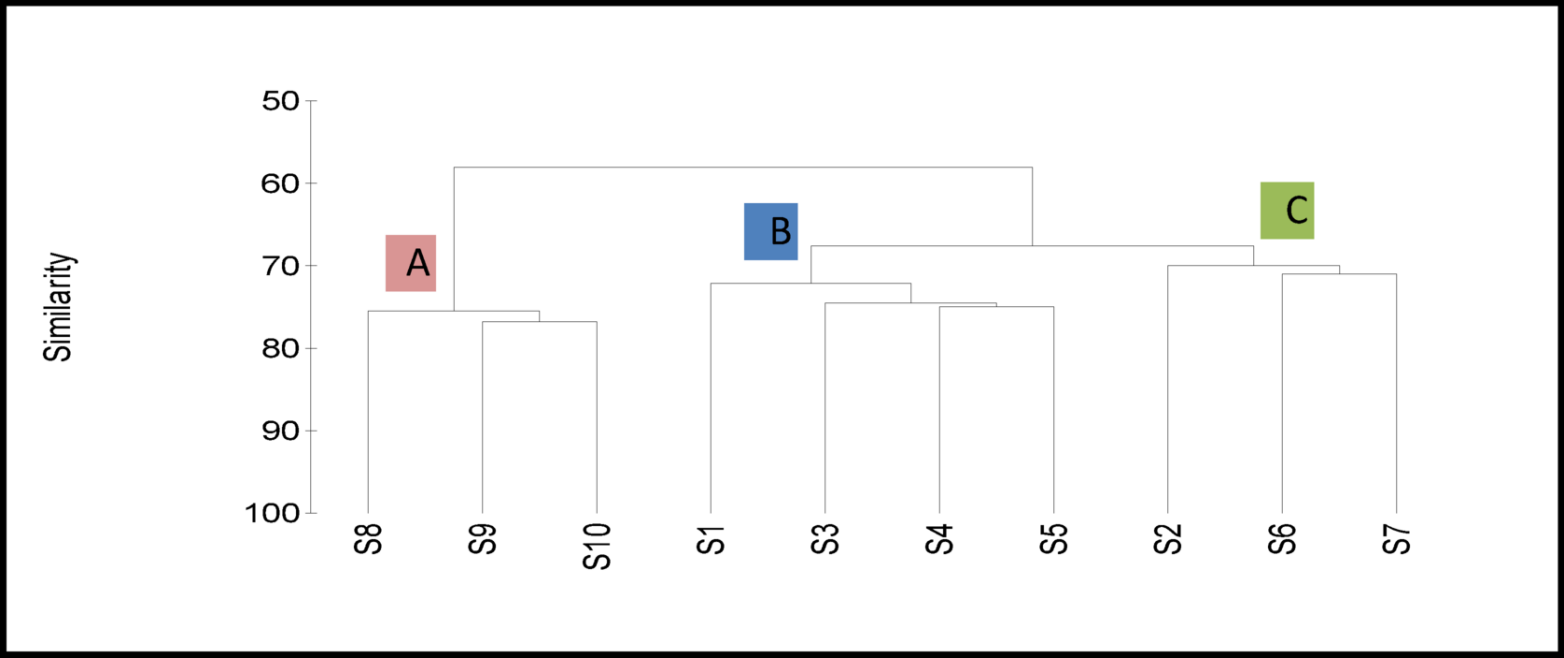
Dendrogram showing similarity among the studied sites in Wadi Mariout Pond according to physicochemical characteristics, bacteriological analysis, phyto- and zooplankton and macrobenthic community structure.

The similarity between the sampling sites based on the physicochemical characteristics, bacterial measurements, phyto- and zooplankton in addition to macrobenthic communities revealed two clusters at about a 58% similarity level (Fig. 10). The first cluster included S8–10 of the western basin (Group A). This group contained the highly polluted sites that agreed with the high CTSI and poor WQI values in addition to the high phytoplankton flourishing and low biodiversity indices of Zooplankton and MBI than all the other sites at the two basins. In particular, S10 showed very poor water quality and higher CTSI and exceeded the allowable limits for fecal streptococci in water with a complete absence of molluscs and Annelida groups. This finding suggested the presence of a different wastewater source affecting this area of the western basin. The second cluster contained all the eastern basin sites (S1–S5) and S6 and S7 at the western basin. The second cluster is divided into two subgroups at a 68% similarity level; the first subgroup (Group B) is comprised mainly of the eastern basin sites (S1, S3, S4, and S5) with a similarity level of about 72% more divided into two mini subgroups where S1 was isolated from the other sites at 73% similarity. This division suggested the presence of a minor wastewater source closer to S1 than the other sites in the eastern basin. The highest similarity was 76% between S4 and S5. The second subgroup (Group C) contained S2, S6 and S7 with 70% similarity. The cluster analysis results correspond with the results of the phyto- and zooplankton distribution and diversity indices (Fig. 10).

## Materials and methods

### Study area

**Fig. 11:**
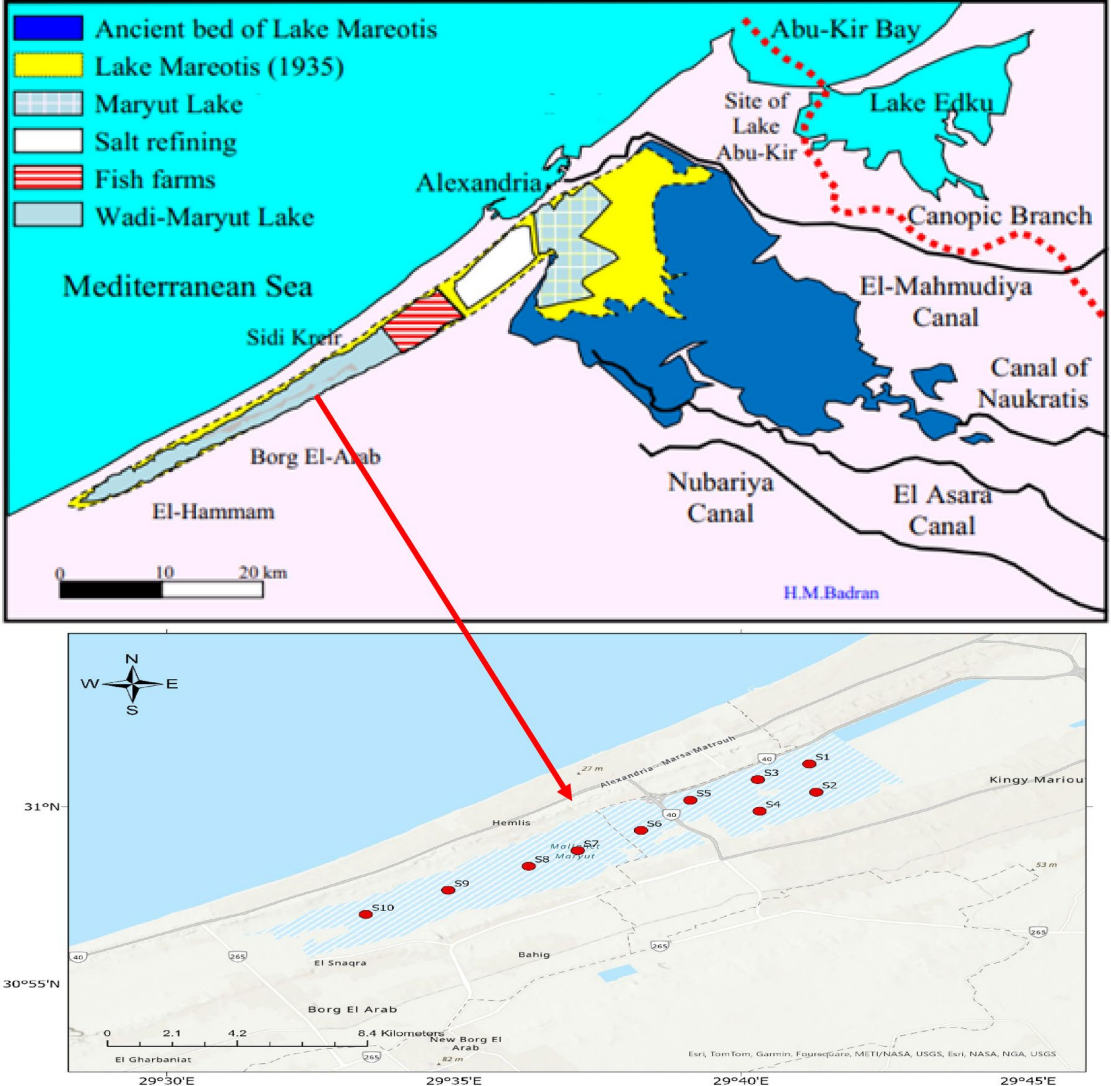
Location sites of the two basins of the Wadi Mariout after Hassan and Badran^45^ , (The map was generated by ArcGis pro 3.1.0, https://experience.arcgis.com/experience/968e183c67774fa786298483a001438d/page/ArcGIS-License-request//).

Wadi Mariout depression is extended about 70 km southwest of Alexandria between Abu-Sir Ridge and Gebel Mariout Ridge^134^. The pond was located in a region that stretched between 30°59′02.7″ to 31°01′47.2″ N latitude and from 29°38′37.5″ to 29°42′24.6″ E longitude (Fig. 11 and Table S5). Numerous causeways, canals, and highways separate it from Mariout Lake’s main body. It measures two to four kilometers wide by fifty kilometers long. An extended island, measuring less than 200 m in width and 4 km in length, is also present. Being fed by rainfall, agricultural runoff, and groundwater seepage, it is a hydrologic basin that is isolated from other bodies of water and has an unpredictable water supply. Most of the year, large portions of this basin are dry^45^. The study area contains two basins: the eastern and western basins. The eastern basin has about 5.5 km of length and 3 km of width, while the western one has 12 km of length and 2.3 km of maximum width.

### Sampling

#### Water

Ten sites from two basins of the Wadi Mariout Pond were selected: five sites (S1–S5) in the eastern basin and five sites (S6–S10) in the western basin. The locations of sampling sites are shown in Table S5 (in the supplementary file) and Fig. 11. Thirty subsurface (about 30 cm) water samples (triplicate from each site) were collected using a Ruttner Water Sampler bottle (2 L) during August 2023. Clean plastic bottles were used for sample storage. For every sample, in situ measurements of temperature, pH, salinity, and EC were made. Samples of biological oxygen demand (BOD) and dissolved oxygen (DO) were carefully put into glass stoppered oxygen bottles and fixed immediately. For bacteriological water analysis, samples were manually collected in sterile dark bottles (200 ml), samples were stored at 4 °C, and bacteriological analysis was completed within 48 h. For the phytoplankton, the water samples were collected and preserved using 4% formalin. For zooplankton samples, thirty liters of water were filtered through a plankton net of mesh diameter 55 μm at each site. The samples were kept in plastic bottles with some lake water, to which 4% formalin was added as a preservative.

#### Sediment

An Ekman grab sampler whose opening area is 440 cm^2^ was utilized to collect sediment and macrobenthic samples duplicate from each site. To conduct a bacterial examination, 10 g of sediment was added to 90 ml of peptone water (0.1% w/v) and shaken for one hour. Afterwards, more serial dilutions were made for further analysis. The macrobenthic samples were collected from the uppermost layer of the sediment bed by passing them through a 500 μm mesh net to exclude any residual debris or particles. Next, the sample was immediately cleaned and placed in polyethylene containers that were marked with the corresponding date. The sample was then mixed with a 10% neutral formaldehyde solution.

### Procedures

#### Water analysis

##### Physicochemical characteristics

The method analysis of APHA^134^ was used to determine the abiotic parameters of the lake water. Water temperature (°C), pH, Salinity (‰), and conductivity (EC, mS / cm) were measured in situ using the Portable Hydrolab multiparameter (Thermo Scientific Orion Star A329). The pH portable meter was calibrated with buffer solutions (4, 7, and 10) before use. Moreover, the EC electrode was calibrated using a standard KCl solution with concentrations of 12.88 mS/cm at 25 °C ± 0.11 mS/cm at 25 °C. Triplicate samples were collected from each site (for physicochemical parameters) and analyzed for nutrients to crosscheck the accuracy of the analytical method, and the mean was computed using relative standard deviations that were below 5%. A white/black Secchi disk (25 cm in diameter) was employed in transparency measurements (SD). The modified Winkler method, the 5-day method, and the potassium permanganate method were used to find DO, BOD, and COD, respectively^134^. Nitrogen-ammonia (N-NH_4_) and nitrogen-nitrite (N-NO_2_) were measured by the phenate and colorimetric azo-dye method^134^, respectively. Nitrogen-nitrate (N-NO_3_) is determined by the cadmium reduction of nitrate to nitrite, followed by the colorimetric azo-dye method. Orthophosphate (P-PO_4_) and silicate (SiO_4_) were estimated using the ascorbic acid molybdate and molybdosilicate methods, respectively. Total Phosphorus (TP) was analyzed by digestion with a strong oxidizing agent in an autoclave at 120 °C for 2 h, followed by the ascorbic acid molybdate method. The detection limits were 1.41 mg/l, 2.1 µg/l, 0.14 µg/l, 0.7 µg/l, 0.93 µg/l, and 3.72 µg/l for SiO_4_^−^, NH_4_^+^, NO_2_^−^, NO_3_^−^, PO_4_^3−^ and TP respectively. The precisions of nutrient salts were estimated by repeat analyses and were within 10% for all nutrients (5–10% for SiO_4_^−^; 4–9% for NH_4_^+^, 3–9% for NO_2_^−^; 2–7% for NO_3_^−^, 2–6% for PO_4_^3−^, and 4–10% for TP). Dissolved inorganic nitrogen (DIN) concentration was computed as the sum of nitrate, nitrite, and ammonium. Carbonate and bicarbonate ions were immediately determined by titration with 0.025 NH_2_SO_4_ utilizing phenolphthalein and methyl orange indicators. Total alkalinity (TA) is the sum of carbonate and bicarbonate alkalinity. Chloride is determined according to Mohr’s method. Sulfate is determined by precipitation with Ba^2+^ using the turbidimetric method. The “EDTA Titrimetric Method” with Eriochrome Black T and Murexide indicators is used to find the calcium and magnesium ions. The flame photometer Model ‘Jenway PFP, U.K.’ is used to measure the levels of sodium and potassium. For chlorophyll-a (Chl-a) analysis, water samples were filtrated using pre-combusted GF/F filters (nominal pore size 0.7 μm). Then the GF/F filters were used for chlorophyll-a extraction using 90% acetone for 24 h in a refrigerator. Under dark conditions, the samples were centrifuged at high speed to obtain the supernatant, which was then measured using double beam UV/visible spectrophotometer (model Jenway 680, United Kingdom) at wavelengths of 750, 630, 647, and 664 after zeroing using a blank sample. Chl-a was calculated according to Jeffrey and Humphrey^135^ by the following equation (Eq. 1):

1 $${Chl}_{a}=\frac{[11.58\times \left({\text{E}}_{664}-{\text{E}}_{750}\right)-1.54\times \left({\text{E}}_{647}-{\text{E}}_{750}\right)-0.08\times \left({\text{E}}_{630}-{\text{E}}_{750}\right)]\times {\text{V}}_{\text{e}}}{\text{L}\times {\text{V}}_{\text{f}}}$$

Where L = cuvette light path in centimeters, V_e_ = volume of extract solvent in ml, V_f_ = volume of sample filtered in liters, and concentrations are in µg/L.

**Carlson Trophic State Index (CTSI)**.

CTSI is an important index to evaluate the eutrophication state of the lakes depending on Seeci Disk (SD) in meters, Chl_a_, and TP concentrations in µg/l, according to Carlson and Simpson^136^. It is calculated by the Eqs. 2–5. The classification of CTSI into values < 30, 30–50, 50–70, and > 70 may refer to oligotrophic, mesotrophic, eutrophic, and hypereutrophic, respectively.

2 $$TSI \: \left(TP\right)=14.42\times Ln\left(TP\right)+ 4.15$$

3 $$TSI\left(SD\right)=60-14.41\times Ln\left(SD\right)$$

4 $$TSI\left({Chl}_{a}\right)=9.81\times Ln\left({Chl}_{a}\right)+ 30.6$$

5 $$\text{C}\text{T}\text{S}\text{I}=\frac{\text{T}\text{S}\text{I}\left(\text{C}\text{D}\right)+\text{T}\text{S}\text{I}\left(\text{T}\text{P}\right)+\text{T}\text{S}\text{I}\left({\text{C}\text{h}\text{l}}_{\text{a}}\right)}{3}$$

**Water Quality Index**.

The arithmetic water quality index (Ar-WQI) is used and computed by Brown et al.^137^ to describe the suitability of the Mariout basin water for aquatic life. The equations, values, and classes of Ar-WQI are in Table 4. Temperature, pH, DO, COD, BOD, NH_4_-N, NO_2_-N, and NO_3_-N are the major variables in calculating the WQI for aquatic life protection.

**Table 4:** The equations, and evaluation criteria involved in calculating WQI.

WQI	Equation	Evaluation criteria	References
Arithmetic	$\text{W}\text{Q}\text{I}=\frac{\sum _{\text{i}=1}^{\text{n}}{\text{Q}}_{\text{i}}{\text{W}}_{\text{i}}}{\sum _{\text{i}=1}^{\text{n}}{\text{W}}_{\text{i}}}$ ${Q}_{i}=\frac{({C}_{i}-{C}_{0})}{({S}_{i}-{C}_{0})}$ ${W}_{i}=\frac{K}{{S}_{i}}$ $K=\frac{1}{\sum _{i=1}^{n}{W}_{i}}$	0 < WQI > 25, Excellent 26 < WQI > 50, Good 51 < WQI > 75, Poor 76 < WQI > 100, Very poor WQI > 100, unsuitable	Brown et al.^137^, Tyagi et al.^138^, Chowdhury et al.^139^ and Balan et al.^140^

Where Q_i_ is the sub-quality index of ith parameter, W = weight unit of each parameter, n = number of parameters, C_o_ = ideal value of ith parameter in pure water, C_o_ = zero for all parameters except for pH = 7.0 and DO = 14.6 mg/l^141^.

##### Biotic parameters

The enumeration of total coliforms (TC), fecal coliforms (FC), and fecal streptococci (FS) utilized the most probable number (MPN) procedure^142^. Specifically, tryptose broth was used for total coliforms (at 37 °C ± 0.5 °C for 48 ± 2 h), EC broth media for fecal coliforms (at 44.5 °C ± 0.5 °C for 24 ± 2 h), and azide dextrose broth for fecal streptococci (at 35 °C ± 0.5 °C for 48 ± 2 h). Moreover, the enumeration of total bacteria (TBC) was performed on Nutrient agar medium utilizing the pour plate method at incubation temperatures of 22 °C and 37 °C^118^. Regarding phytoplankton analysis, preserved water samples (with 4% formalin) were subjected to qualitative and quantitative examination. Species identification and counting were done using a light microscope employing the Utermöhl method^142^, with phytoplankton abundances expressed as Ind.l^−1^. The phytoplankton community was identified at species levels^143,144^. Additionally, the biochemical parameters of the separated phytoplankton, including total protein, carbohydrate, and lipid content, were meticulously determined. The total protein content was evaluated using the Biuret method^145^, carbohydrate contents were measured according to the phenol sulfuric acid method as described by Dubois et al.^146^, and total lipid content was determined by the sulfo-phospho-vanillin procedure (SPV)^147^. Subsequently, concentrations of proteins, carbohydrates, and lipids were converted to biopolymeric carbon (BPC) as per Fabiano et al.^148^. Furthermore, zooplankton samples were examined under a compound microscope, and when feasible, specimens were identified at the species level. Zooplankton density was quantified as the number of organisms per cubic meter according to Tahoun et al.^149^.

#### Sediment analysis

The sediment samples are being prepared according to the methods outlined by Folk^150^ and analyzed using the Fritsch laser size analyzer analyst 22 Nano Tec with a dry dispersion unit, which covers a range from 2 mm to 100 nm. Organic matter in the sediment is determined using the loss on ignition method described by Hanna^151^. Water content analysis is conducted according to the procedures outlined by Baruah and Barthakur^152^.

Total bacteria (TBC) are enumerated on nutrient agar medium at incubation temperatures of 22 and 37 °C using the pour plate method^151^. Suitable dilutions are pour-plated on Sorbitol MacConkey Agar (SMAC) (Lab M Limited, UK). After 24 h of incubation at 35 °C, colonies with a typical red or colorless/gray appearance with a smoky centre are counted as total coliforms, while only the colorless/grey colonies with a smoky center are considered fecal coliforms.

The macrobenthic samples underwent a secondary round of washing and sieving using a net with a mesh diameter of 0.5 mm. The benthic animals were then sorted into their respective genera or species using a zoom stereo microscope, and each group was counted. Each species was carefully preserved in a glass container with a 7% formalin solution for identification purposes. The density of benthic invertebrates was measured as the number of individuals per square meter. The specimens were meticulously examined and identified in the laboratory, with precise references to all relevant macrobenthic sources used for identification. The sample collection, preparation, and preservation procedures followed the methods outlined in El-Shabrawy et al.^153^.

### Statistical analysis

The descriptive statistics, such as the minimum, maximum, mean, and standard deviation values, are expressed using the Minitab^®^ statistical program version 18.1. To demonstrate the homogeneity of the data, Anderson-Darling’s normality test was performed. An unpaired sample t-test was employed to compare the examined variables between the two basins of the Wadi Mariout Pond at *P* < 0.05. The associations between the studied variables in the pond water were examined using the Pearson correlation coefficient. Using STATISTICA 10 (StatSoft Inc. 2011), bacterial data were statistically examined using analysis of variance (ANOVA). To compare means at *P* < 0.05, the Fisher LSD test was employed as a post hoc test. To relate the phytoplankton, zooplankton, and macrobenthos results with the water and sediment parameters, canonical correlation analysis (CCA) was used for some of the parameters. Using the PAST4.03 program, the biodiversity indices of Shannon–Winner, Margalef (species richness), and Equitability (species evenness) were computed. The findings were verified by Shannon^154^, Margalef^82^, and Pielou^81^.

## Conclusion

This study comprehensively presents the first scientific data profile to identify the abiotic and biotic features of Wadi Mariout Pond. This study outlines the ecological and taxonomical aspects of aquatic phytoplanktons, zooplanktons, and macrobenthic invertebrates inhabiting Wadi Mariout Pond in addition to their correlation with the water physicochemical characteristics, bacterial counts, and sediment grain size analysis. The physicochemical variables referred to brackish water with significant differences in salinity between the two basins (15.37–16‰ and 12.28–14.06‰) for the eastern and western basins, respectively. The arithmetic water quality index (WQI) referred to excellent water quality at 100% of the samples of the eastern basin. On the other hand, the western basin exhibited good, poor, and very poor water quality at 60, 20, and 20% of water samples, respectively. However, CTSI indicated a eutrophic state, with an average mean value of 63.52 and 64.61 for the eastern and western basins, respectively, which refers to high ecological productivity. The CCA investigates the relations between the investigated species and the chemical parameters. The CCA results in water referred to salinity, temperature, DO, NO_3_, NO_2_, pH, and BOD as the most important factors that positively affected the cyanobacteria, *Chroococcus* spp., Dinophyta, and *Gyrodinium* sp. and negatively associated with most taxa of zooplankton such as *Brachionus* spp., *Hexarthra mira*, Nauplius larva, Copepoda, and total zooplankton in addition to total bacteria counts at 37 °C and 22 °C and fecal coliform (FC). Moreover, COD greatly influenced the green algae (*Botryococcus* sp.) and the Dinophyta species (*Prorocentrum donghaiense*). Also, depth, PO_4_, Na, K, and SiO_2_ had a big effect on Chlorophyta, Bacillariophyta, and their dominant species (*Chlorella sorokiniana* and *Cyclotella ocellata*). Where phytoprotein represents the building block of the biochemical contents (≈ 90%), followed by carbohydrates (≈ 8%) and lipids (≈ 1.5%). Also, The CCA date referred to salinity, DO, pH, OM, and water temperature as the most ecological factors that significantly affected the benthic macroinvertebrate community. Therefore, the hydrography, nutrients, and sediment texture are the major factors responsible for fluctuation in the benthic macrofauna assemblages in the study area. The one-way cluster analysis results referred to S8, S9, and S10 as the major hot spots in the western basin and agreed with the CTSI and WQI data, bacteriological analysis, and highly affected phyto-zooplankton, and macrobenthic community structures. Furthermore, they referred to S1 as the minor hot spot directly affected by wastewater at the eastern basin. More research in the field of molecular biology at Wadi Mariout Pond is needed to provide more information about the microalgae involved in the production and recycling of carbon in the body of the studied water environment. The findings indicate the need to develop plans to control the quality of water sources for the Wadi Mariout pond to reduce pollution and prevent their transformation into a highly polluted water body as happened in Lake Mariout, accompanied by certain mitigating measures. Additionally, establishing a comprehensive geographic information system (GIS) for the region is crucial for detecting changes and facilitating decision-making.

## Electronic supplementary material

Below is the link to the electronic supplementary material.

Supplementary Material 1

## Data Availability

All data generated or analyzed during this study are included in this published article [and its supplementary information files].
