# Supplementary material for: A comprehensive evaluation of the ecological status of Wadi Mariout ponds, Egypt
Source: Sci Rep. 2025 Apr 30;15:15256. doi: 10.1038/s41598-025-97129-6 (PMC12043857; doi:10.1038/s41598-025-97129-6)
Supplement: Supplementary file 1 — Supplementary Material 1 [file 41598_2025_97129_MOESM1_ESM.docx]

**Supplementary File**

**A Comprehensive evaluation of the ecological status of Wadi Mariout ponds, Egypt**

Alaa I. Khedr^1^, Mohamed H. Abdo^1^, Radwan G. Abd Ellah^1^, Shaimaa M. Ibrahim^1^, Eman I. Abdel-Aal^1^, Howayda H. Abd El-Hady^1^, Nehad Khalifa^1^, Heba E. EL-Sebaie^1^, Amal A. Othman^1^, Salem G. Salem^1^, Mohamed E. Goher^1^*

^1^**National Institute of Oceanography and Fisheries, NIOF, Cairo, Egypt**

Figure S1: Temperature variations at the selected sites in the Wadi Mariout Pond.

Figure S2: EC variations at the selected sites in the Wadi Mariout Pond.

Figure S3: Variations of total suspended solids (TSS) at the selected sites in the Wadi Mariout Pond.

Figure S4: Salinity variations at the selected sites in the Wadi Mariout Pond.

Figure S5: pH variations at the selected sites in the Wadi Mariout Pond.

Figure S6: DO variations at the selected sites in the Wadi Mariout Pond.

Figure S7: Variations of COD at the selected sites in the Wadi Mariout Pond.

Figure S8: Variations of BOD at the selected sites in the Wadi Mariout Pond.

Figure S9: variations of total alkalinity (TA) at the selected sites in the Wadi Mariout Pond.

Figure S10 variations of ammonia (µg/l) at the selected sites in the Wadi Mariout Pond.

Figure S11: variations of nitrite (µg/l) at the selected sites in the Wadi Mariout Pond.

Figure S12: variations of nitrate (µg/l) at the selected sites in the Wadi Mariout Pond.

Figure S13: variations of orthophosphate (µg/l) at the selected sites in the Wadi Mariout Pond.

Figure S14: variations of silicate (mg/l) at the selected sites in the Wadi Mariout Pond.

Figure S15: variations of TP (µg/l) at the selected sites in the Wadi Mariout Pond.

Figure S16: variations of Chl a (µg/l) at the selected sites in the Wadi Mariout Pond.


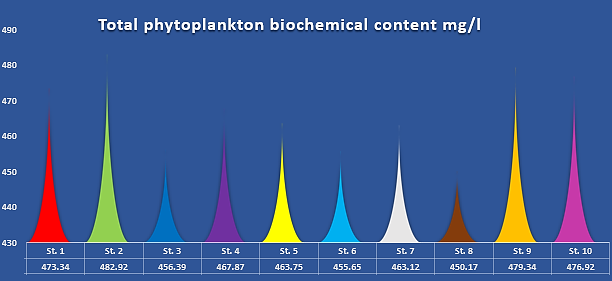


**Fig. S17.** Nutritive value of phytoplankton at selected sites of Wadi Mariout Pond.

**Table S1:** Trophic State Classification Scheme based on the CTSI values.

| Parameter | Oligotrophic | Mesotrophic | Eutrophic | Hyper-Eutrophic | Study Area | | Status |
| --- | --- | --- | --- | --- | --- | --- | --- |
|  |  |  |  |  | **Eastern basin** | **Western basin** |  |
| TP (µg/l) | 0-12 | 12—24 | 24-96 | 96-384+ | 66.3-93.05 | 42.92-69.49 | **Eutrophic** |
| Chl a (µg/l) | 0-2.6 | 2.6-7.3 | 7.3-56 | 56-155+ | 10.48-12.97 | 8.33-28.11 | **Eutrophic** |
| SD (m) | 4-8 | 2-4 | 0.5-2 | <0.25-0.5 | 0.9-1.15 | 0.8-1.3 | **Eutrophic** |
| TSI | 0-40 | 40-50 | 50-70 | 70-100 | 62.93-63.99 | 61.31-67.88 | **Eutrophic** |
| WQ | Good | Fair | Poor | Very Poor | Poor | Poor |  |
| N:P ratio |  |  |  |  | N- limiting | N and P colimited |  |

**Table S2:** Spatial changes in bacterial indicator of pollution along Wadi Mariout water ponds (ANOVA analysis).

| **Sites** | **Total coliform (MPN/100) F(9,20) = 2.0253, *p*= 0.091** | **Fecal coliform (MPN/100)** | **Fecal streptococci (log no./100) F(9,20)=12684, *p*= 0.000*** |
| --- | --- | --- | --- |
| **S 1** | 9 ^ab^ | 0 | 2.4^d^ |
| **S 2** | 0 | 0 | 3.04^b^ |
| **S 3** | 0 | 0 | 3.04^b^ |
| **S 4** | 12 ^a^ | 0 | 3.04^b^ |
| **S 5** | 12 ^a^ | 3 | 3.04^b^ |
| **S 6** | 5 ^ab^ | 0 | 3.04^b^ |
| **S 7** | 4 ^ab^ | 3 | 2.66^c^ |
| **S 8** | 12 ^a^ | 0 | 2.66^c^ |
| **S 9** | 4 ^ab^ | 0 | 2.66^c^ |
| **S 10** | 5 ^ab^ | 0 | 5.08 ^a^ |

ANOVA: means followed by the same letter are not significantly different (P < 0.05).

**Table S3:** Presence and absence of the recorded phyto-, zooplankton and MBI species in the studied sites of two basins in Wadi Mariout ponds.

|  | **Eastern Basin** | **Western Basin** |
| --- | --- | --- |
| **Phytoplankton** |  |  |
| **Cyanophyta** |  |  |
| *Aphanocapsa delicatissima* | - | 1 |
| *Chroococcus dispersus* | 5 | 5 |
| *Chroococcus dispersus var. minor* | 3 | 5 |
| *Chroococcus minutus* | 3 | 5 |
| *Chroococcus turgidus* | 2 | 3 |
| *Coelosphaerium kuetzingianum* | - | 2 |
| *Merismopedia glauca* | 4 | 3 |
| *Gomphosphaeria aponina* | 1 | - |
| *Merismopedia punctata* | - | 3 |
| *Synechococcus* sp | 3 | 5 |
| **Chlorophyta** |  |  |
| *Botryococcus* sp | 5 | 5 |
| *Chlamydomonas globosa* | 2 | 4 |
| *Chlorella sorokiniana* | 5 | 5 |
| *Cosmarium sp* | 1 | - |
| *Crucigeniella quadrata* | 2 | - |
| *Dunaliella sp* | 1 | 1 |
| *Pyramimonas* sp | - | 3 |
| *Kirchneriella lunaris* | 2 | 3 |
| *Monoraphidium contortum* | - | 1 |
| *Oocystis borgei* | 1 | 2 |
| *Oocystis elliptica* | 1 | - |
| *Scenedsmus* sp | 1 | 1 |
| **Bacillariophyta** |  |  |
| *Cocconeis placentula var. placentula* | 2 | - |
| *Chaetoceros sp* | 1 | 2 |
| *Cyclotella ocellata* | 5 | 5 |
| *Navicula muralis* | 3 | 5 |
| *Navicula* sp. | - | 1 |
| *Nitzschia* sp | 1 | 1 |
| *Thalassiosira* sp | 3 | 5 |
| *Detonula* sp | 5 | 5 |
| **Dinophyta** |  |  |
| *Gymnodinium* sp | 1 | 1 |
| *Gyrodinium* sp | 3 | 5 |
| *Prorocentrum donghaiense* | 3 | 4 |
| *Prorocentrum gracile* | - | 1 |
| **Cryptophyta** |  |  |
| *Cryptomonads* sp | 5 | 5 |
| *Rhodomonas* sp. | - | 4 |
| **Crysophyta** |  |  |
| *Ochromonas* sp. | 2 | 1 |
| **Zooplankton** |  |  |
| R**otifera** |  |  |
| *Anuraeopsis fissa* | 3 | 1 |
| *Asplanchnella sieboldi* | 1 | 4 |
| *Brachionus angularis* | 3 | 2 |
| *Brachionus calyciflorus* | 1 | 2 |
| *Brachionus leydigii* | - | 4 |
| *Brachionus plicatilis* | 5 | 5 |
| *Brachionus quadridentatus* | 5 | 5 |
| *Brachionus rubens* | 2 | - |
| *Brachionus urceolaris* | 5 | 5 |
| *Epiphanes macroura* | 3 | 3 |
| *Hexarthra mira* | 5 | 5 |
| *Keratella tropica* | 2 | 1 |
| *Lecan monostyla* | - | 1 |
| *Polyarthra vulgaris* | 4 | 4 |
| **Copepoda** |  |  |
| Cyclopoid copepod | 5 | 5 |
| Nauplius larva | 5 | 5 |
| **Cladocera** |  |  |
| *Diaphanosoma excisum* | 1 | - |
| **Prortozoa** |  |  |
| *Turborotalita humilis* | 3 | 2 |
| *Acanthocystis aculeata* | 1 | - |
| *Arcella spp.* | - | 1 |
| **Macrobenthos** |  |  |
| **Arthropoda** |  |  |
| Chironomidae larva | 5 | 3 |
| Chironomidae pupa | 3 | - |
| *Gammarus aequicauda* | 1 | - |
| *Corophium volutator* | 1 | - |
| **Mollusca** |  |  |
| *Venerupsis aurea* | 4 | 1 |
| *Cerastoderma glaucum* | 5 | 4 |
| *Mytilus* sp. | 4 | 2 |
| **Annelida** |  |  |
| *Ficopomatus enigmaticus* | 2 | - |
| *Nereis diversicolor* | 1 | - |
| Tubificidae sp. | 5 | 2 |

+ : present; –: absent; number beside+: number of sites.

**Table S4:** Spatial changes in bacterial indicator of pollution along Wadi Mariout sediment (ANOVA analysis).

| **Sites** | **Total coliform (cfu/g)** | **Fecal coliform (cfu/g)** | **Fecal streptococci (log no./g)** |
| --- | --- | --- | --- |
|  | **F(9, 20)=371.04, *p*=0.000*** | **F(9, 20)=335.67, *p*=0.000*** | **F(9, 20)=9.1972, *p*=0.000*** |
| **S 1** | 26 ^c^ | 0 | 3.36 ^f^ |
| **S 2** | 18 ^c^ | 1 ^d^ | 4.45 ^cd^ |
| **S 3** | 18 ^c^ | 0 | 4.39 ^de^ |
| **S 4** | 3384 ^a^ | 150 ^a^ | 4.83 ^b^ |
| **S 5** | 252 ^b^ | 23^b^ | 4.28 ^e^ |
| **S 6** | 33 ^c^ | 0 | 4.51 ^c^ |
| **S 7** | 16 ^c^ | 10 ^c^ | 4.94 ^b^ |
| **S 8** | 15 ^c^ | 0 | 4.45 ^cd^ |
| **S 9** | 15 ^c^ | 2 ^d^ | 4.50 ^cd^ |
| **S 10** | 15 ^c^ | 0 | 5.87 ^a^ |

ANOVA: means followed by the same letter are not significantly different (P < 0.05).

**Table S5:** GPs reading of location sites of Wadi Mariout Ponds

| Site | Lat N | Long E |
| --- | --- | --- |
| S1 | 31° 1'12.67" | 29°41'11.70" |
| S2 | 31° 0'24.87" | 29°41'18.83" |
| S3 | 31° 0'46.13" | 29°40'17.91" |
| S4 | 30°59'52.63" | 29°40'19.85" |
| S5 | 31° 0'11.25" | 29°39'7.10" |
| S6 | 30°59'19.96" | 29°38'15.63" |
| S7 | 30°58'45.88" | 29°37'9.73" |
| S8 | 30°58'19.44" | 29°36'18.29" |
| S9 | 30°57'38.46" | 29°34'54.44" |
| S10 | 30°56'57.59" | 29°33'28.20" |

.
